# From Self-Worth to Sustainable Choice: An Efficacy-Translation Account of Green Consumption

**DOI:** 10.3390/bs16091477

**Published:** 2026-08-25

**Authors:** Tao Jiang, Fengpei Hu, Xiadan Yang, Ming Guo

**Affiliations:** 1 Center for German-speaking Country Studies, Zhejiang University of Science and Technology, Hangzhou 310023, China; 2 School of Management, Zhejiang University of Science and Technology, Hangzhou 310023, China; 3 School of Management, Zhejiang University of Technology, Hangzhou 310000, China; fengpei@zjut.edu.cn (F.H.); 51214410003@stu.ecnu.edu.cn (X.Y.); 4 School of Asia Europe Business, East China Normal University, Shanghai 200241, China

**Keywords:** self-esteem, green consumption, perceived consumer effectiveness, sustainable consumption, attitude–behavior gap, computational behavioral audit

## Abstract

Green consumption research has long documented a gap between favorable environmental attitudes and concrete sustainable choices. Yet less is known about why consumers who value sustainability sometimes doubt that their own actions can matter. Across five studies of Chinese consumer samples (*N* = 1499), this article develops an efficacy-translation account of green consumption. The account proposes that global self-esteem is associated with perceived consumer effectiveness (PCE), the belief that individual consumption can contribute to environmental improvement. It therefore specifies PCE as a domain-specific collective-impact belief rather than as a generic efficacy construct. The evidence incorporates trait and state self-esteem, measured and manipulated PCE, and multiple green-consumption outcomes, validated by robust regression, bootstrap mediation, internal meta-analysis, specification-curve analysis, and a limited computational audit. Self-esteem was positively associated with green consumption across the five studies, with a random effects pooled association of r=0.164, 95% CI [0.114, 0.213]. In Study 2A, the measured indirect association through PCE was 0.105, 95% bootstrap CI [0.048, 0.164]. These results are consistent with PCE as one pathway linking self-worth to sustainable consumption, but they do not establish causal mediation or generalize beyond the sampled cultural context.

## 1. Introduction

Green consumption has become a central behavioral response to environmental degradation, climate risk, and the growing social expectation that consumers should participate in sustainability transitions. Consumers often express favorable attitudes toward environmentally responsible products, but these attitudes do not always become concrete choices, willingness to pay, or repeated behavior (Duong, 2021; Nguyen et al., 2019; Peattie, 2010). This attitude–behavior gap is not merely a measurement inconvenience. It is a theoretical problem for behavioral science because it implies that environmental concern, moral approval, and pro-environmental intention are insufficient to explain when consumers act.

Prior research has explained the gap through product price, availability, social norms, environmental values, and consumer knowledge (Biswas & Roy, 2015; Coderoni & Perito, 2020; Hanss et al., 2016). Policy signals and perceived value also shape whether favorable attitudes become green choices (Ng et al., 2023; Yang et al., 2023; Yaqub et al., 2024). These accounts clarify why green products are often not chosen in the real world. Yet they leave a psychological question underexplored: how do consumers’ evaluations of themselves shape perceived environmental impact? A consumer may value sustainability and still feel that an individual purchase is too small to matter. In such cases, the barrier is not indifference but perceived inefficacy.

This distinction matters for theory and for the interpretation of possible interventions. If the attitude–behavior gap is mainly an information problem, then education should be central. If it is mainly a cost problem, then subsidies and product redesign should dominate. If it partly reflects perceived consumer effectiveness, messages that make personal impact credible may merit testing. The present data do not test interventions or establish that such messages change behavior.

This article develops an efficacy-translation account of green consumption. In this account, self-esteem is a global evaluation of personal worth (Rosenberg, 1965). PCE is a domain-specific belief that one’s consumption efforts can help address environmental or social problems (Ellen et al., 1991; Jaiswal & Kant, 2018; Kinnear et al., 1974). We use psychological interface as a descriptive term for the proposed link between global self-evaluation and a collective-impact belief. The account predicts an association between self-esteem and PCE, and between PCE and green consumption. It does not assume that PCE is the sole or proven causal route.

The account contributes in three bounded ways. First, it asks whether a global self-evaluation covaries with a more proximal, consumption-specific collective-impact belief, rather than treating self-esteem and PCE as parallel predictors. This differs from general self-efficacy, which concerns broad capability, and perceived behavioral control, which concerns perceived ease or control over a specified behavior. Environmental identity, moral self-concept, and prosocial motivation concern identification or reasons for action. PCE instead concerns the expected environmental consequence of one’s consumption. The present data do not measure all adjacent constructs, so they cannot establish PCE’s unique empirical status relative to them. They do, however, generate a discriminating prediction: if PCE is merely a correlated proximal predictor, self-esteem should add no reproducible information to PCE or green consumption once common orientation variables are considered; if the account is useful, self-esteem should show a positive association with PCE and a measured indirect association with green consumption. Second, the measurement gradient design tests whether the association is visible across intention, trade-off choice, and past behavior. Third, a limited computational audit evaluates incremental information beyond attitude, value, knowledge, and demographic baselines. The intended advance is a bounded, culturally situated account, not a universal mechanism.

The paper therefore treats computation as an evidentiary aid for behavioral theory. The computational layer is included because recent behavioral science work increasingly emphasizes transparent, reproducible, and heterogeneity-aware analysis. Predictive modeling is used conservatively. It is not a prediction-first contribution. It neither replaces theory nor serves as a substantive contribution. Its role is to test whether the theoretical constructs remain informative in a richer behavioral profile.

The resulting design links three forms of evidence. Mechanism analyses test whether the observed associations are consistent with the proposed account. The measurement gradient asks whether the association remains visible as outcomes move from intention to choice and past behavior. The computational audit asks whether self-esteem and PCE retain incremental information after stronger behavioral baselines are included.

This framing also constrains the limits of the paper’s contribution: the manuscript proposes a bounded mechanism, not a universal predictor, and evaluates that mechanism against alternative explanations before drawing substantive implications.

## 2. Related Work and Hypothesis Development

### 2.1. Green Consumption and the Attitude–Behavior Gap

Green consumption refers to purchasing, using, and disposing of products in ways that reduce environmental harm and support long-term collective welfare (Carlson & Kangun, 1993; Peattie, 2010). The field has moved beyond simple demographic profiling toward psychological, social, and contextual explanations of sustainable behavior. Environmental values, subjective norms, green product attitudes, perceived value, product attributes, and policy contexts all shape consumer decisions (Biswas & Roy, 2015; Hanss et al., 2016; Ng et al., 2023). Recent 2024–2026 evidence continues this shift. Studies of pandemic-era sustainable consumption and Generation Z show that environmental knowledge is most consequential when paired with perceived consumer effectiveness (Choi et al., 2025; Minh & Nguyen, 2024). Biodiversity-friendly consumption research and a recent meta-analysis likewise identify PCE as an action-enabling construct rather than a peripheral attitude variable (Uusitalo et al., 2026; Vieira et al., 2025). Digital literacy research links green food consumption to attitudes and perceived behavioral control rather than information access alone (J. He et al., 2025).

These recent studies show that knowledge and values rarely act alone. They become behaviorally consequential when consumers perceive themselves as capable of converting concern into effective action. The present contribution builds on this direction but changes the theoretical level of analysis. Instead of asking whether PCE is another strong predictor, it asks how self-worth is translated into PCE and whether that translation remains visible across different forms of green consumption measurement.

The attitude–behavior gap remains a persistent concern. Consumers may express strong environmental concern yet choose conventional products when green alternatives are costly, inconvenient, or uncertain (Duong, 2021; Nguyen et al., 2019). This gap has encouraged research on perceived behavioral control, green trust, social influence, and situational constraints (X. He & Zhan, 2018; Liang et al., 2020; Xie & Madni, 2023). However, much of this work treats the consumer as already motivated and asks what blocks action. The efficacy-translation account adds a self-evaluative layer: consumers also differ in whether they believe their own actions are consequential.

The gap is also methodological. Many studies rely on a single intention scale, which can inflate apparent support for green consumption. Stronger evidence requires multiple outcome forms that vary in psychological cost and behavioral specificity. In the present dataset, general intention is the broadest and least costly outcome, multi-category green-versus-conventional choice introduces a trade-off, and past green behavior is closer to realized action. A mechanism that appears across this gradient is more persuasive than one that appears only in abstract intention.

### 2.2. Self-Esteem as a Psychological Resource

Self-esteem captures global self-worth and the extent to which individuals see themselves as valuable and capable (Branden, 1969; Rosenberg, 1965). It has trait and state components: trait self-esteem is relatively stable, whereas state self-esteem can fluctuate in response to situational cues, success, failure, and social feedback (Paradise & Kernis, 2002; Patrick et al., 2004). In consumer contexts, self-esteem influences self-verifying consumption, status consumption, and reactions to products that signal identity or competence (Souiden & Pons, 2011; Stuppy et al., 2020).

Self-esteem is also relevant to prosocial behavior. People with higher self-esteem are more likely to believe they can contribute to others and to engage in cooperative or helping behavior, although these effects depend on context and threat (Jami et al., 2021; Moscardino et al., 2020; Sun et al., 2021). Green consumption is not only a market act; it is also a prosocial and moralized act because its benefits often accrue to broader society rather than only to the purchaser (Griskevicius et al., 2010). Self-esteem should therefore increase green consumption only when consumers view their own actions as capable of making a difference.

This qualification is important. A simple self-esteem account would predict that high self-esteem consumers should always be greener. That claim is too broad for the present evidence and for the structure of green consumption behavior. We therefore treat the self-esteem association as a baseline prediction. The primary theoretical question is whether its association with green consumption is statistically consistent with a pathway through PCE.

**Hypothesis 1.**  *Self-esteem positively predicts green consumption.*

### 2.3. Perceived Consumer Effectiveness as Efficacy Translation

PCE is central to environmental consumer research because it reflects whether consumers believe individual action can contribute to environmental improvement (Ellen et al., 1991; Kinnear et al., 1974). It differs from general environmental concern because a consumer can care deeply about climate or pollution while doubting that a single purchase matters. It differs from general self-efficacy because it is not a broad capability judgment. It also differs from perceived behavioral control because it concerns the consequence of a consumption act, not simply whether that act is easy to perform. These are conceptual distinctions, not claims of discriminant validity in the present data. A 2025 meta-analysis identifies PCE and environmental concern as robust green behavior predictors (Vieira et al., 2025), and recent studies link PCE to socially responsible and biodiversity-friendly action (Choi et al., 2025; Uusitalo et al., 2026).

The proposed account is that PCE may statistically link self-esteem to green consumption. Higher self-esteem may be associated with a greater tendency to view oneself as an effective agent. That tendency may covary with willingness to choose greener products, accept trade-offs, and report sustainable behavior. In the measured mediation study, a reduced direct coefficient after PCE is included is compatible with this account, but it is not evidence of full mediation or causal transmission.

The translation metaphor clarifies how this article differs from studies that simply add PCE as another predictor. The account specifies a directional theoretical ordering, from global self-evaluation to a consumption-specific collective-impact belief. The data can assess whether this ordering is consistent with the observed pattern. They cannot, without experimental manipulation of self-esteem or PCE in the mediation design and measurement of adjacent constructs, establish the ordering as the only causal explanation.

**Hypothesis 2.**  *PCE mediates the relationship between self-esteem and green consumption.*

### 2.4. Issue Importance and Heterogeneity

The perceived importance of an ethical issue shapes moral judgment and behavioral intention (Chan et al., 2008; Haines et al., 2008; Robin et al., 1996). In green consumption, high issue importance may motivate action even among consumers who have lower self-esteem, because the issue itself supplies motivational force. When issue importance is low, however, consumers may rely more heavily on self-related psychological resources to determine whether action is worthwhile.

This reasoning implies a possible boundary to the pathway. Self-esteem should matter most when green consumption is not already perceived as highly important. Under high issue importance, concern and moral salience may compress individual differences in self-esteem. Under low issue importance, self-esteem and PCE should become more diagnostic of who acts.

This boundary condition has possible intervention implications, but the current evidence base is better suited to exploratory boundary probing than to a decisive moderation test. Importance messages and efficacy messages are often combined in environmental communication, but they may not be equally useful for all consumers. If issue importance is already high, additional severity or importance messaging may be redundant. If issue importance is low, efficacy framing may help consumers see green consumption as a domain in which their own choices have consequence. We therefore treat issue importance as a theory-generating boundary question to be evaluated conservatively and preregistered in future intervention work.

Exploratory Research Question 1. Is the association between self-esteem and green consumption stronger when perceived issue importance is lower?

Exploratory Research Question 2. Is the indirect pathway from self-esteem to green consumption through PCE numerically larger when perceived issue importance is lower?

### 2.5. Computational Audit as Behavioral Science Method

Recent consumer research increasingly uses computational methods to examine heterogeneity, improve transparency, and evaluate whether theoretical variables add explanatory value beyond conventional controls (Athey & Wager, 2019; Mullainathan & Spiess, 2017; Puntoni et al., 2021). In the present research, the computational audit is secondary to the regression and ablation analyses. Its sole additional role is to compare out-of-sample performance of transparent linear models with a flexible non-linear benchmark. This comparison cannot adjudicate causal structure. With N=300 in Study 3, it may also favor regularized linear models because the random forest has limited data for estimating interactions. Detailed benchmark and permutation-importance outputs are therefore reported in Supplementary Materials S1.

The reference to a future incentive-compatible design has been moved to Limitations and Future Research.

## 3. Materials and Methods

### 3.1. Data Harmonization and Reproducibility Audit

The source surveys were harmonized into a common analytic structure before hypothesis testing. The final dataset contains five studies and 1499 valid observations. The resulting evidence base supported reliability assessment, robust regression, bootstrap mediation, internal meta-analysis, specification-curve analysis, and predictive-validity benchmarking.

The harmonization step was treated as part of the validity audit rather than as a clerical operation. For each study, the audit evaluated variable definitions, missing-value patterns, unique values, means, standard deviations, and observed ranges. This step made the evidentiary chain traceable and confirmed that the five-study analytic sample contained 1499 valid observations.

### 3.2. Confirmatory Status, Sample Scope, and Ethics Documentation

The five studies were not preregistered. Hypotheses 1 and 2 were theory derived but should therefore be interpreted as non-preregistered tests, not as confirmatory preregistered tests. The issue-importance analyses were exploratory throughout. All samples were recruited from Chinese consumers, and claims are limited accordingly. All five studies were conducted within the stated protocol approval period of 19 October 2025 to 18 October 2026: Study 1A on 6 June 2026, Study 1B on 8 June 2026, Study 2A on 11 June 2026, Study 2B on 15 June 2026, and Study 3 on 18 June 2026. Each date is the reported recruitment and data-collection date for that study.

### 3.3. Study Overview

Table 1 summarizes the five studies, their green-consumption outcomes, and their roles in testing the proposed account.

### 3.4. Measures

Self-esteem was operationalized with a Chinese-language rendering of the ten Rosenberg Self-Esteem Scale items in each measurement study (Rosenberg, 1965). The archival materials identify Rosenberg (1965) as the item source but do not document a separate validated Chinese translation or adaptation protocol; we therefore do not claim use of a specific validated translation. The Study 1A data export contains all ten items, including the four items marked as reverse-worded; the exported composite score was computed over the ten item-level variables. Study 1B also used a success/failure recall task to vary state self-evaluation. PCE was measured with five items on whether individual consumption could contribute to environmental improvement, including one reverse-scored item, following Vermeir and Verbeke (2006). Green consumption was operationalized as general intention, past self-reported behavior, or an eight-category green-versus-conventional trade-off index. The eight categories were automobiles, light bulbs, mobile phones, air conditioners, gasoline, laundry detergent, milk packaging, and printing paper. The archival materials do not document a separate rationale for selecting these eight categories. The paired options differed in both environmental attribute and price: conventional/green prices were RMB 100,000/150,000 per automobile, 8/15 per bulb, 1200/2000 per phone, 2300/3400 per air conditioner, 6.42/7.92 per litre of gasoline, 25/35 per container of detergent, 3/5 per bottle of milk, and 20/35 per pack of printing paper. The choices therefore combine environmental attributes with monetary trade-offs; the materials did not include separate familiarity ratings or environmental-impact ratings. The exact translated stimuli are provided in Supplementary Materials S1, the original Chinese questionnaire in Supplementary Materials S2, and the English measurement materials in Supplementary Materials S3. Perceived issue importance was measured with four semantic-differential items in Study 3.

Control variables included gender, age, education, income, marital status, residence, green product attitude, and green consumption value. These controls test whether self-esteem and PCE explain variance beyond consumers’ general environmental orientation.

The study-level reliability pattern was inspected before hypothesis testing. Green value scales were consistently reliable, and the eight-category choice measures showed strong internal consistency. With all ten Study 1A self-esteem items included, the lowest self-esteem coefficient was α=0.702 in Study 2A, and the lowest PCE coefficient was α=0.622 in Study 3. Lower reliability can attenuate observed associations, so the resulting small coefficients may be conservative. A disattenuation sensitivity analysis is reported in Supplementary Materials S1; it is descriptive only and does not remove other sources of bias. Individual coefficients are interpreted cautiously, with emphasis on convergence across studies, bootstrap intervals, and the internal meta-analysis.

### 3.5. Statistical Models

For the main effect, each study was analyzed using robust OLS models with HC3 standard errors. Partial correlations between self-esteem and green consumption were calculated after demographic controls only. The HC3 standardized coefficients reported in the study-level results table instead come from the fully adjusted models, which additionally control for green attitude and green values. These estimands answer different questions and are reported for transparency, not as duplicate significance tests. The five partial correlations were synthesized using an internal random-effects meta-analysis based on Fisher’s *z* transformation.

To reduce dependence on a single preferred control specification, the analysis added a compact specification-curve audit. Specification curves and multiverse analyses are useful when behavioral findings may be sensitive to defensible analytic choices (Simmons et al., 2011; Simonsohn et al., 2020; Steegen et al., 2016). For each study, the self-esteem effect was re-estimated under four pre-specified control sets: no controls, demographic controls, green-orientation controls, and demographic plus green-orientation controls. This audit was not used to search for a stronger result; it was used to test whether the direction and inference of the main association were stable across reasonable specifications.

For mediation, Study 2A was analyzed using standardized variables and nonparametric bootstrap resampling with 5000 iterations. The indirect effect was computed as the product of the self-esteem-to-PCE path and the PCE-to-green-consumption path. HC3 robust standard errors were used for the component paths and total/direct effects. Residual diagnostics indicated heteroskedasticity and non-normality in these regressions; the robust and bootstrap procedures address sampling inference but do not verify the causal assumptions of cross-sectional mediation. Because self-esteem, PCE, and past behavior were measured variables in Study 2A, this analysis estimates an indirect association under an assumed ordering; it does not identify a causal mediation effect. For exploratory boundary analysis, Study 3 tested self-esteem, issue importance, and their interaction in predicting PCE and green consumption.

The planned ablation sequence compares four nested model families. The first includes only demographic controls. The second adds green attitude and green value, which are strong conventional predictors in green-consumption research. The third adds self-esteem, testing whether self-worth explains residual variance beyond established green orientations. The fourth adds PCE and, where available, issue importance and interaction terms. This design prevents the manuscript from overstating self-esteem as a standalone predictor and instead evaluates whether the proposed translation mechanism adds theoretically meaningful explanatory value.

Three safeguards were built into the analytic design. First, all regression tests used the same harmonized individual-level records rather than manually transcribed summary tables. Second, the key claim was evaluated at the study level and then synthesized across studies. This design prevented one unusually favorable sample from dominating the manuscript. Third, the computational audit separated explanatory interpretation from predictive adequacy. A variable was considered theoretically useful only when it had a clear conceptual role and improved the nested model or interpretable audit. This is why PII is treated as exploratory even though it is useful in the prediction profile.

### 3.6. Computational Analysis Procedure

The secondary computational audit was conducted in Study 3. Linear regression, Ridge, Lasso, Elastic Net, and random forest models predicted green-consumption intention from self-esteem, PCE, issue importance, green attitude, green values, moral knowledge items, and demographic controls. All selected variables were converted to numeric form and complete cases were used; all 300 Study 3 records were complete for this feature set. Standardization was performed inside each cross-validation pipeline. Shuffled five-fold cross-validation evaluated predictive adequacy. Ridge, Lasso, and Elastic Net tuning was performed within their specified regularization grids; the random forest used a fixed reproducible specification rather than a broad search. Exact grids, seeds, and model settings are reported in Supplementary Materials S1. Ridge, Lasso, and Elastic Net served as regularized linear baselines (Hoerl & Kennard, 1970; Tibshirani, 1996; Zou & Hastie, 2005) and random forest served as a flexible benchmark (Breiman, 2001). This separation of explanatory and out-of-sample predictive evidence follows recent behavioral-science guidance (Almaatouq & Na, 2026; Plonsky et al., 2025). The audit complements, but does not replace, the more interpretable nested ablation analysis. Table 2 maps the three stated contributions to their corresponding reproducible analyses.

## 4. Results

The results are organized as a converging-evidence test of the efficacy-translation account. The first evidence block asks whether self-esteem is reliably associated with green consumption across outcome forms. The second asks whether PCE carries this association. The third asks whether issue importance and consumer profiles qualify the pathway. The fourth uses interpretable prediction and nested ablation to test whether the theory remains informative within a broader behavioral profile.

### 4.1. Study-Specific Evidence

Study 1A tested whether trait self-esteem predicted green consumption under concrete product trade-offs. Participants evaluated eight product categories in which greener options were paired with conventional alternatives. This design is stricter than a general intention measure because it asks participants to express preference under product-level choice constraints. After controlling for demographic variables, green attitude, and green values, self-esteem remained positively associated with the eight-category green-choice index.

Study 1B tested the same theoretical direction using state self-esteem activation. Participants completed a success or failure recall task designed to temporarily shift state self-evaluation before reporting green-consumption intention. The manipulation-based design provides stronger directional evidence than a purely correlational survey. The self-esteem effect was again positive, indicating that the link between self-evaluation and green consumption is not limited to stable trait differences.

Study 2A examined measured self-esteem, PCE, and past green-consumption behavior. The standardized bootstrap indirect association was positive and excluded zero. The direct coefficient of self-esteem was small after PCE was included. This pattern is consistent with, but does not establish, the proposed efficacy-translation account because the design does not manipulate the predictor or mediator.

Study 2B used the same sample of 300 participants for the original simple-slope analysis and the HC3 robust re-estimation. The latter is an alternative specification, not an independent replication. The control condition read a neutral passage about the historical limitations of information-storage media. The low-PCE condition read a passage emphasizing that individual environmental actions have very small effects and that people may feel powerless to solve environmental problems. The complete translated texts are supplied in Supplementary Materials S1, and the updated original Credamo export is supplied in Supplementary Materials S2. The manipulation check was strong: the control group reported higher PCE than the low-PCE group, d=1.21, p<0.001. The self-esteem slope was positive in the control condition (b=0.058, 95% CI [0.022, 0.095]) and not distinguishable from zero in the low-PCE condition (b=0.012, 95% CI [−0.044, 0.067]). However, the between-condition interaction was not conventionally significant (b=−0.044, 95% CI [−0.099, 0.011], p=0.115). The interval includes both zero and a moderately negative interaction, so the study is too imprecise to support equivalence or to rule out meaningful attenuation. No prospective interaction-specific power analysis was available. Conditional on the observed HC3 standard error of approximately 0.028, a two-sided test at α=0.05 would require an interaction of about |b|=0.079 for approximately 80% power; thus, the study was not sufficiently precise to resolve small interaction effects. The difference between a significant and a non-significant simple slope is not itself evidence of moderation. Study 2B is therefore inconclusive for the predicted interaction and is retained only as a manipulation check with suggestive attenuation evidence. The study did not include separate familiarity, environmental impact, affect, or motivation checks. Green consumption values and green product attitude were measured after the randomized passage and differed between conditions, so the manipulation cannot be treated as exclusively PCE-specific.

Study 3 tested perceived issue importance as an exploratory boundary condition. The theoretical expectation is that self-esteem should matter more when green consumption is not already perceived as highly important. The reproducibility audit showed the expected numerical pattern for conditional indirect effects, but the standardized interaction terms were weak across robust specifications. This pattern is therefore reported as exploratory boundary evidence rather than as a settled moderation claim.

Table 3 summarizes the study-level main-effect evidence.

### 4.2. Reliability and Measurement Quality

Most multi-item measures showed acceptable reliability. Green product attitude and green value scales were consistently acceptable to strong, and eight-category green choice measures showed high reliability. The lower coefficients were Study 2A self-esteem (α=0.702) and Study 3 PCE (α=0.622). They can attenuate correlations and do not justify treating any single-study coefficient as definitive. A descriptive correction-for-attenuation sensitivity analysis is reported in Supplementary Materials S1, while the main interpretation relies on cross-study convergence rather than corrected effects. Table 4 summarizes reliability across the core multi-item constructs.

### 4.3. Self-Esteem and Green Consumption

**Figure 1:**
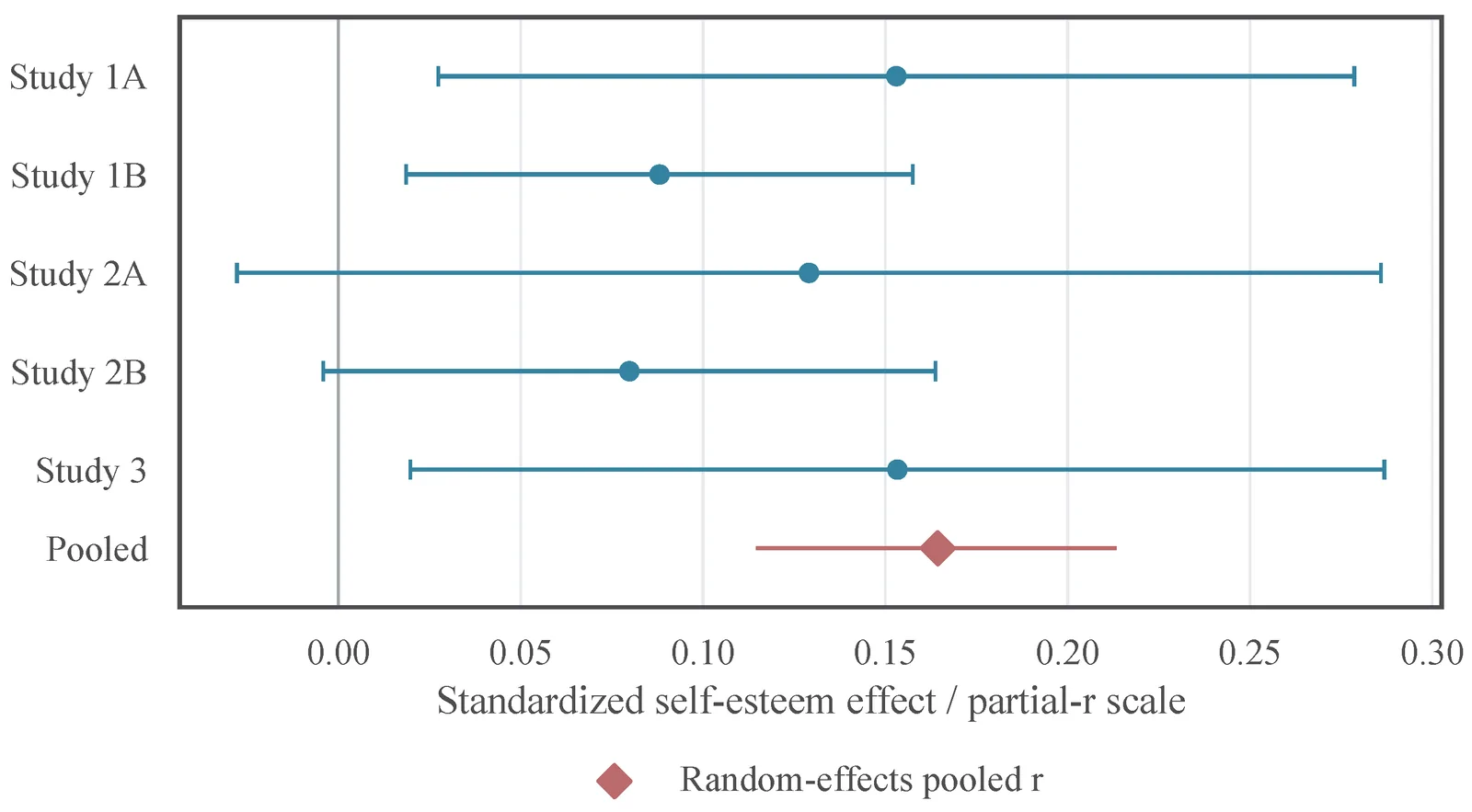
Internal robustness of the self-esteem effect across five studies. The figure summarizes robust study-level estimates and the pooled random-effects association; blue circles denote study-level partial correlations, and the red diamond denotes the pooled random-effects estimate. Legend is placed below the figure in the source graphic.

Self-esteem positively predicted green consumption across all five studies after controlling for demographic variables, green product attitude, and green values. Partial correlations ranged from r=0.137 to r=0.186, all in the predicted direction. The random-effects meta-analysis yielded a pooled association of r=0.164, 95% CI [0.114, 0.213], Q=0.409, $I^{2}=0\%$. This pattern supports a prerequisite for the account: the self-worth signal was modest, but it appeared across intention, trade-off choice, and past-behavior evidence (Figure 1).

Leave-one-study-out checks supported the same conclusion. Dropping any single study yielded pooled associations from r=0.159 to r=0.171, and heterogeneity remained $I^{2}=0\%$ in each re-estimation. This matters because the five studies differ in self-esteem evidence, outcome type, and mechanism measurement. The stability of the pooled estimate therefore supports the measurement gradient claim: the association is not an artifact of one intention scale, one manipulated study, or one behavior-proximal outcome.

**Figure 2:**
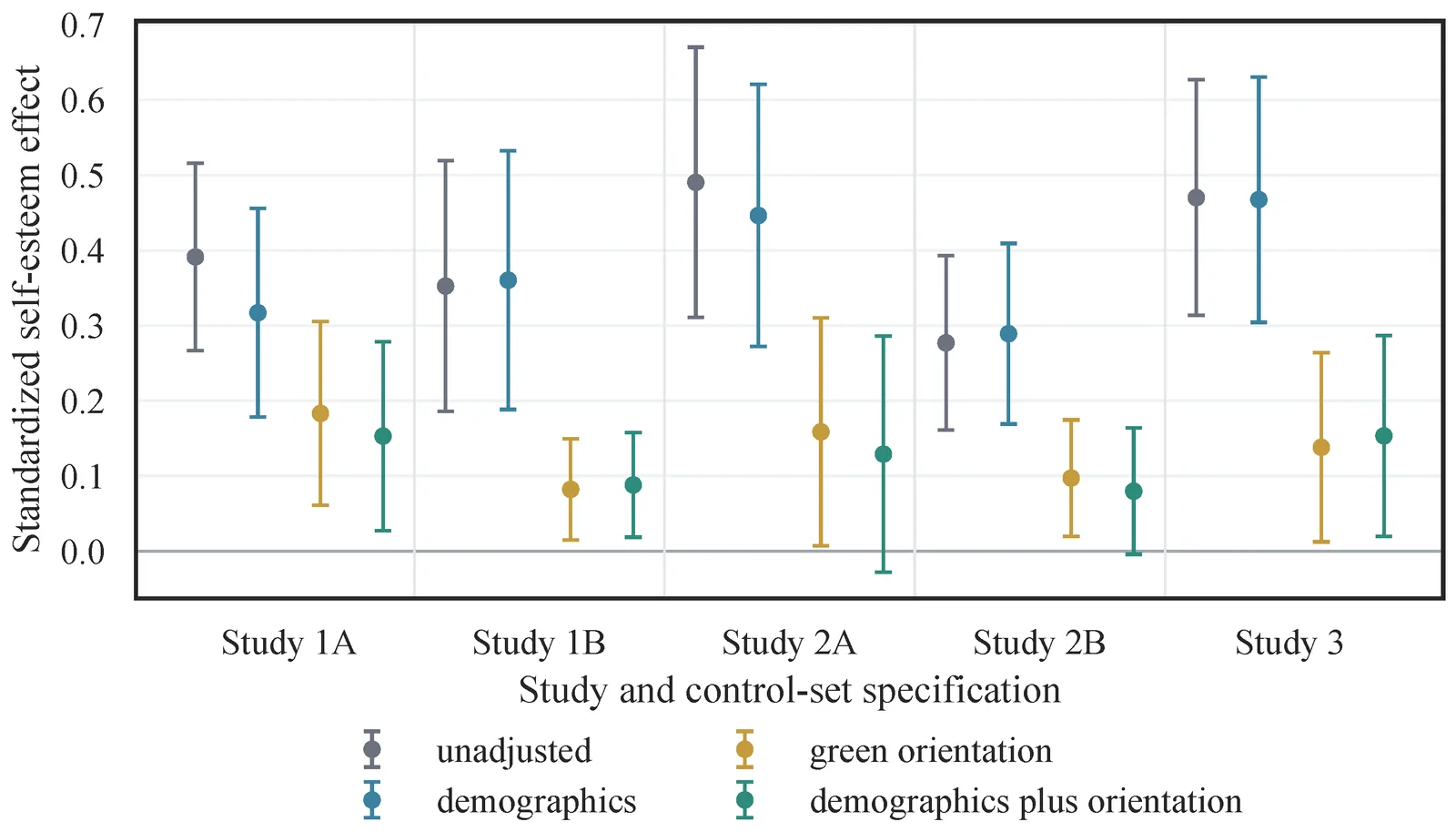
Specification-curve audit of the self-esteem effect. Each point represents one study-by-control-set model with HC3 standard errors. The audit shows that the association remained positive across unadjusted, demographic-adjusted, green-orientation-adjusted, and fully adjusted specifications.

The specification-curve audit further reduced the risk that the main conclusion depended on one analytic specification. Across 20 study-by-control-set specifications, all self-esteem coefficients were positive, and 18 of 20 were conventionally significant at p<0.05. The median standardized self-esteem coefficient was 0.230, with coefficients ranging from 0.080 to 0.490 across the multiverse of control choices. The two non-significant specifications occurred in Study 2A and Study 2B under the most restrictive demographic-plus-orientation controls, but both remained positive. This pattern, summarized in Figure 2, supports a bounded robustness claim: the self-esteem association is not large, but its direction is not an artifact of a single selected model.

### 4.4. PCE as the Efficacy Translation Mechanism

**Figure 3:**
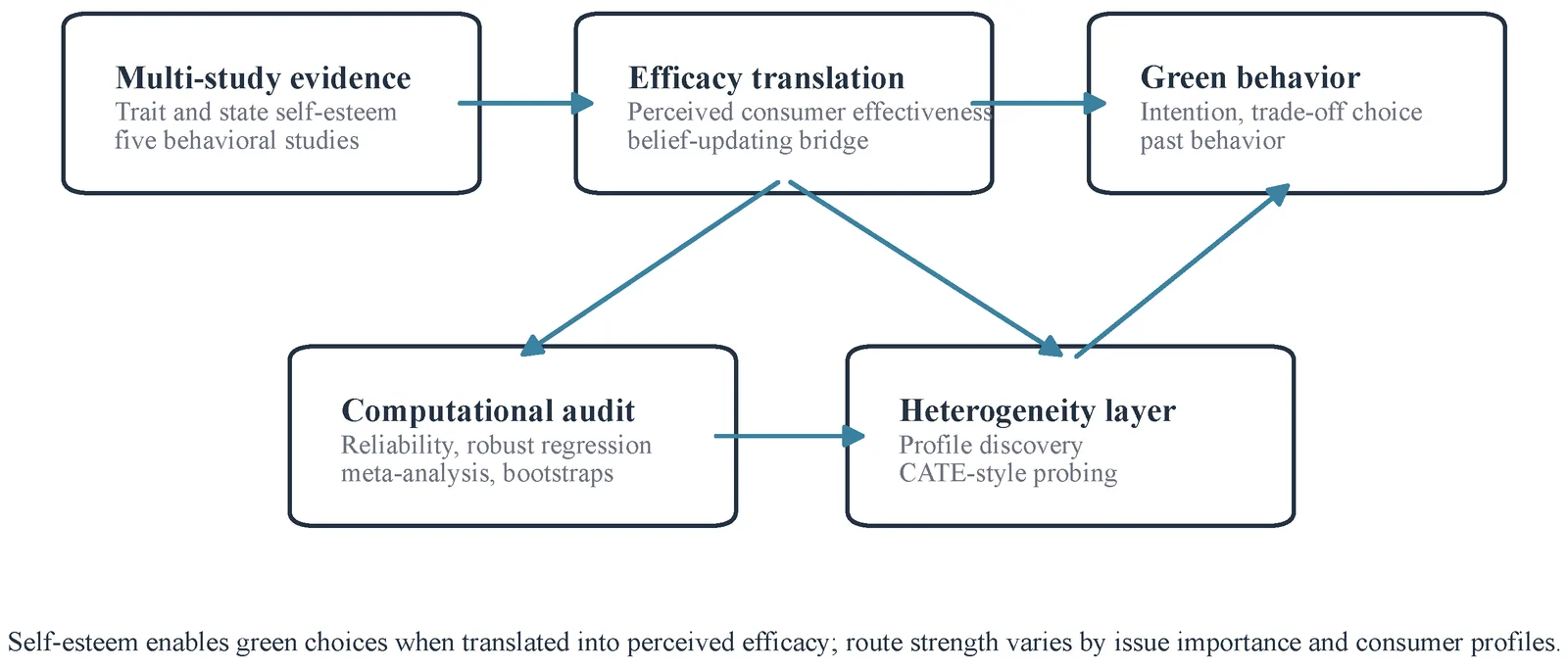
Efficacy-translation framework and audit layers. The self-esteem-to-PCE-to-green-consumption path is a theoretical ordering tested with measured mediation; it is not a causal diagram. The heterogeneity layer is exploratory and does not imply a supported direct pathway to green behavior.

Study 2A yielded a measured indirect association consistent with the proposed account. In standardized mediation with demographic, attitude, and value controls, self-esteem predicted PCE (a=0.343, HC3 SE = 0.066), and PCE predicted green consumption conditional on self-esteem (b=0.305, HC3 SE = 0.069). The total self-esteem association was c=0.129 (HC3 SE = 0.080, p=0.107); the direct coefficient after adding PCE was $c^{\prime }=0.024$ (HC3 SE = 0.071). The indirect association was 0.105, 95% bootstrap CI [0.048, 0.164]. Because all three variables were measured rather than experimentally manipulated, the model does not establish the causal assumptions required for mediation and should not be read as full mediation or causal transmission. Figure 3 summarizes the theoretical ordering and the evidentiary layers.

Table 5 summarizes the Study 2B manipulation check, conditional slopes, and interaction estimate.

The table clarifies why Study 2B is not a successful test of the predicted interaction. The manipulation produced a large reduction in PCE, but it also coincided with higher raw green attitude and green values in the low-PCE condition. The interaction confidence interval includes zero. The simple-slope pattern is reported descriptively and not used as evidence that the slopes differ. The supplied materials also show that product prices differed systematically between conventional and green options, so the choice index combines environmental attributes with cost trade-offs.

### 4.5. Issue Importance and Consumer Heterogeneity

Study 3 provided no statistically reliable evidence that perceived issue importance moderated the self-esteem association. Across full, truncated, and winsorized specifications, the interaction was non-significant. We therefore report this analysis only as an exploratory null result; full sensitivity analyses and the descriptive tertile plot have been moved to Supplementary Materials S1.

### 4.6. Computational Audit

The computational audit is reported briefly because nested ablation provides the more directly interpretable test of incremental information. In five-fold cross-validation, Ridge achieved $R^{2}=0.497$ and random forest achieved $R^{2}=0.428$. This result does not show that the psychological structure is linear or that random forest failed. The sample size and predictor count may limit the flexible model’s ability to estimate non-linearities. Full benchmark and permutation-importance results are in Supplementary Materials S1. Table 6 reports the nested ablation audit.

The nested ablation table gives the computational audit a direct behavioral science interpretation. Attitude, value, and knowledge variables explain the largest share of variance, which is expected and prevents exaggeration of the findings. Self-esteem nevertheless adds incremental explanatory value beyond this conventional baseline ($\Delta R^{2}=0.015$). PCE adds a larger increment ($\Delta R^{2}=0.029$), and issue-importance/profile variables add further information ($\Delta R^{2}=0.027$). The cross-validated pattern is similar: adding self-esteem, PCE, and PII/profile information improves out-of-sample $R^{2}$ and reduces MAE. Thus, the computational audit supports the theory as a bounded incremental mechanism rather than a dominant prediction engine.

### 4.7. Ablation and Robustness Logic

The main robustness logic is comparative rather than decorative. A demographics-only model establishes the minimal baseline. Adding green attitude and green values tests whether the model captures established predictors in sustainable-consumption research. Adding self-esteem tests whether self-worth has incremental value beyond those predictors. Adding PCE tests whether PCE adds incremental information after self-esteem and conventional predictors. This sequence does not establish causal interpretation. Table 7 states the review function of each model family.

Table 8 consolidates the principal quantitative evidence from the reproducibility audit.

## 5. Discussion

Across five studies, self-esteem showed a modest, directionally consistent association with green consumption across general intention, trade-off choice, and past behavior. The size of the pooled association (r=0.164) means that self-esteem is not a dominant determinant of sustainable consumption. Its theoretical relevance is limited to a possible self-related correlate of PCE to behavior that is also shaped by price, values, norms, attitudes, and product attributes.

The central contribution is the efficacy-translation account. It does not portray self-esteem as a generic trait of green consumers or identify a single causal mechanism. Instead, it specifies a theoretical order in which global self-worth is associated with a consumption-specific belief that one’s actions can help. The measured mediation and incremental analyses are consistent with that ordering, while adjacent constructs and alternative causal orders remain plausible.

This contribution differs from a conventional antecedent model because it gives PCE a specified conceptual role. Attitude and values describe motivational orientation, PCE describes perceived action consequence, and self-esteem is a global self-evaluation. The current evidence cannot determine whether PCE is uniquely distinct from self-efficacy or perceived behavioral control, nor can it rule out confounding or reverse causality. Future work should measure these constructs together and manipulate the proposed mediator.

The measurement-gradient design is the second substantive contribution. The evidence spans abstract intention, multi-category green-versus-conventional choice, and past green behavior. The internal meta-analysis and leave-one-study-out checks show bounded reproducibility rather than broad claims of strong behavioral determination.

The third contribution is methodological. The paper combines a measurement gradient, robust standard errors, bootstrap inference, internal meta-analysis, and sensitivity analyses. These procedures improve transparency but do not eliminate common-method bias, causal-identification limits, or the risks associated with non-preregistered analyses.

The computational audit is included as a transparent supplement to the ablation analysis, not as an independent technical contribution. Its out-of-sample comparison indicates that, in this sample, regularized linear models performed better than the selected random-forest specification. That result is compatible with many explanations, including limited sample size for a flexible model, and does not identify psychological structure.

### Theoretical Implications

The findings extend the literature on PCE by treating it as a theoretically specified link rather than only a direct predictor. Consumers high in PCE are more likely to endorse green purchases, carbon labels, and sustainable apparel (Choi et al., 2025; Liang et al., 2020). The present work asks whether global self-worth is one correlate of PCE. It does not show that self-esteem creates PCE or that PCE is the only efficacy-related explanation.

The findings also refine self-esteem theory in consumer contexts. Green consumption is not primarily about displaying superiority or verifying identity; it depends on whether a consumer experiences the self as efficacious in a collective-action setting. The effects of self-esteem on consumer behavior may therefore depend on whether the market action allows agency.

Finally, the results contribute to attitude–behavior-gap research by identifying perceived action consequence as a plausible correlate that merits stronger causal tests. The present data do not support profile-targeted communication recommendations, because the issue-importance moderation was non-significant and no intervention was tested. Any practical use should therefore await preregistered experimental evidence with consequential choices.

This positioning also clarifies the manuscript’s methodological boundary. The evidence should not be read as a commercial application or product-design study. Its contribution is a behavioral mechanism supported by computationally transparent evidence, with interpretable profiling and heterogeneity analysis used only to constrain the theoretical claims.

## 6. Conclusions

This research develops a bounded efficacy-translation account of green consumption in Chinese consumer samples. Across five studies, self-esteem showed a modest association with green consumption, and measured mediation was consistent with PCE as one possible pathway. The evidence does not establish causal mediation, a unique efficacy mechanism, or a generalizable intervention effect.

## 7. Limitations and Future Research

Several limitations qualify the interpretation. First, some analyses depend on self-reported outcomes, and future research should include field purchases or incentive-compatible choice. A laboratory-choice extension could offer a real bonus allocation between a conventional product, a green product, or a verified environmental donation.

Second, the issue-importance interaction was non-significant across the reported robustness specifications. The descriptive pattern should not be treated as evidence of motivational saturation. A future preregistered study could manipulate issue importance and efficacy messages orthogonally to test that conjecture.

Third, the current data are from Chinese consumer samples; cross-cultural replication would strengthen generalizability. Self-esteem, agency, and collective environmental responsibility may operate differently across individualistic and collectivistic contexts.

Fourth, the computational audit is diagnostic rather than causal. The fact that Ridge and Elastic Net outperformed random forest does not identify causal priority among predictors or prove a linear psychological structure. Future work could use preregistered heterogeneous-treatment-effect models only after random assignment to efficacy, importance, and control messages.

Fifth, profile-matched efficacy messaging remains a future behavioral experiment, not current evidence. A definitive future study would randomize generic importance, generic efficacy, and profile-matched efficacy messages with an incentive-compatible green choice as the target outcome. 

## Figures and Tables

**Table 1:** Overview of the five-study measurement-gradient design.

Study	*N*	Self-Esteem Evidence	Green-Consumption Outcome	Mechanism or Boundary Role
1A	300	Trait self-esteem scale	Eight-category green versus conventional product choices	Main effect under concrete trade-off choice
1B	299	State self-esteem manipulation	General green-consumption intention	Causal evidence for self-esteem activation
2A	300	Trait self-esteem scale	Past green-consumption behavior	PCE mediation test
2B	300	Trait self-esteem plus PCE manipulation	Eight-category green versus conventional product choices	Strong manipulation check; inconclusive interaction test
3	300	Trait self-esteem scale	General green-consumption intention	PCE and exploratory perceived issue importance boundary audit

**Table 2:** Evidence map linking the three innovations to reproducible analyses.

Innovation	Operationalization and Audit Evidence	Evidence Produced
Self-worth-to-efficacy translation	Study 2A bootstrap mediation; Study 2B manipulation check	Tests whether self-esteem enters green consumption through PCE and whether PCE can be experimentally shifted.
Measurement-gradient robustness	Five-study HC3 models; Fisher-*z* random-effects meta-analysis; leave-one-study-out and specification-curve checks	Tests whether the association survives intention, trade-off choice, past-behavior outcomes, and alternative control sets.
Computational behavioral audit	Nested ablation, regularized baselines, random forest comparison, permutation importance	Tests whether self-esteem and PCE retain explanatory value beyond attitudes, values, issue importance, knowledge, and demographics.

**Table 3:** Study-level main-effect evidence. Partial *r* controls demographic variables only; HC3 standardized β comes from the fully adjusted model controlling demographic variables, green attitude, and green values. The columns are distinct estimands, not duplicate significance tests.

Study	*N* and Outcome Type	Partial *r*	HC3 Standardized β	Interpretation
1A	300; eight-category trade-off choice	0.172, p=0.003	0.153, p=0.017	Positive association under a concrete trade-off outcome.
1B	299; general intention	0.137, p=0.018	0.088, p=0.013	Positive association after state self-evaluation activation.
2A	300; past behavior	0.167, p=0.004	0.129, p=0.107	Measured mediation is reported separately and cautiously.
2B	300; eight-category trade-off choice	0.159, p=0.006	0.080, p=0.062	Fully adjusted association is positive but not conventionally significant.
3	300; general intention	0.186, p=0.001	0.153, p=0.024	Positive association; boundary analysis is exploratory.

**Table 4:** Reliability summary for core multi-item constructs.

Construct	Studies with Available Scale	Reliability Range	Manuscript Implication
Self-esteem	1A, 1B, 2A, 2B, 3	α≈0.70–0.93	Effects should be interpreted through cross-study convergence rather than any single scale.
PCE	2A, 2B, 3	α≈0.62–0.93	Study 2B shows strong manipulation-check reliability; Study 3 requires cautious interpretation.
Green intention	1B, 3	α≈0.70–0.76	General intention outcomes are acceptable but should not stand alone.
Past green behavior	2A	α=0.702	Retrospective self-reported behavior is more behavior-proximal than intention but remains subject to recall and reporting bias.
Green trade-off choice	1A, 2B	α≈0.87–0.94	Multi-category choice is the strongest outcome reliability evidence.
Green product attitude	1A, 1B, 2A, 2B, 3	α≈0.69–0.93	Attitudes are primary orientation controls and should be modeled alongside green values.
Green values	1A, 1B, 2A, 2B, 3	α≈0.87–0.94	Values are strong controls and should remain in all main models.

**Table 5:** Study 2B PCE manipulation and attenuation pattern.

Evidence	Control	Low-PCE	*p*	Effect
PCE manipulation check	5.536	3.887	<0.001	d=1.212
Raw green-choice mean	4.930	5.642	<0.001	d=−0.516
Self-esteem slope, 95% CI	0.058 [0.022, 0.095]	0.012 [−0.044, 0.067]	0.001/0.680	Suggestive attenuation only
SE × low-PCE interaction	–	–	0.115	b=−0.044, 95% CI [−0.099, 0.011]

**Table 6:** Nested ablation audit in Study 3.

Model Family	OLS $R^{2}$	$\Delta R^{2}$	CV $R^{2}$	CV MAE
Demographics	0.056	–	−0.032	0.416
+Attitude/value/knowledge	0.528	0.473	0.424	0.300
+Self-esteem	0.543	0.015	0.441	0.296
+PCE	0.572	0.029	0.462	0.291
+PII/profile	0.599	0.027	0.502	0.284

**Table 7:** Ablation logic used to evaluate incremental theoretical value.

Model Family	Included Predictors	Review Function
Demographics baseline	Gender, age, education, income, marital status, residence	Rules out simple socio-demographic explanation.
Attitude/value baseline	Demographics, green attitude, green values	Tests against dominant green-consumption predictors.
Self-esteem increment	Baseline predictors plus self-esteem	Evaluates whether self-worth explains residual behavioral variation.
Efficacy-translation model	Baseline predictors, self-esteem, PCE	Tests whether PCE adds incremental information under the proposed ordering.
Boundary and profile model	Full model plus issue importance, interactions, and interpretable profiling audit	Evaluates exploratory heterogeneity without treating the boundary as confirmed.

**Table 8:** Main quantitative evidence from the reproducibility audit.

Evidence Block	Estimate	Interpretation
Five-study meta-analysis	r=0.164, 95% CI [0.114, 0.213], $I^{2}=0\%$	Self-esteem has a modest but stable association with green consumption across outcome types.
Study 2A mediation	Indirect association = 0.105, 95% bootstrap CI [0.048, 0.164]	Pattern is consistent with, but does not establish, a PCE pathway.
Study 3 model audit	Best CV $R^{2}=0.497$ with Ridge	Secondary descriptive benchmark; not evidence of causal or linear structure.
Nested ablation	PCE adds $\Delta R^{2}=0.029$ after self-esteem and conventional predictors	The efficacy construct has incremental value beyond attitudes, values, knowledge, and demographics.
Measurement gradient	Intention, trade-off choice, past behavior	The mechanism is not limited to a single intention measure.

## Data Availability

The data presented in this study are available on request from the corresponding author due to ethical and privacy restrictions. The analysis materials and aggregate robustness tables are available in Supplementary Materials S1.

## Supplementary Materials

The following supporting information can be downloaded at https://www.mdpi.com/article/10.3390/bs16091477/s1: Supplementary Materials S1, reproducibility materials, robustness analyses, statistical details, and English translations of the participant-facing Study 2B stimuli; Supplementary Materials S2, the original participant-facing Chinese-language Credamo questionnaire for Study 2B; Supplementary Materials S3, English translations of the Study 2B measurement materials. The Chinese-language content in S2 is retained to document the questionnaire exactly as administered; corresponding English translations are provided in S1 and S3.

## References

[B1-behavsci-16-01477] Almaatouq A., Na R. (2026). The integration of explanation and prediction in behavioral science. Current Directions in Psychological Science.

[B2-behavsci-16-01477] Athey S., Wager S. (2019). Estimating treatment effects with causal forests: An application. Observational Studies.

[B3-behavsci-16-01477] Biswas A., Roy M. (2015). Green products: An exploratory study on the consumer behaviour in emerging economies of the East. Journal of Cleaner Production.

[B4-behavsci-16-01477] Branden N. (1969). The psychology of self-esteem.

[B5-behavsci-16-01477] Breiman L. (2001). Random forests. Machine Learning.

[B6-behavsci-16-01477] Carlson L., Kangun N. (1993). A content analysis of environmental advertising claims: A matrix method approach. Journal of Advertising.

[B7-behavsci-16-01477] Chan R. Y. K., Wong Y. H., Leung T. K. P. (2008). Applying ethical concepts to the study of green consumer behavior. Journal of Business Ethics.

[B8-behavsci-16-01477] Choi L. S. L., Chan K. W., Fock H. (2025). Deepening exploration of socially responsible consumption in Generation Z: The impacts of environmental knowledge and perceived consumer effectiveness. Journal of Consumer Marketing.

[B9-behavsci-16-01477] Coderoni S., Perito M. A. (2020). Sustainable consumption in the circular economy: Consumers’ purchase intentions for waste-to-value food. Journal of Cleaner Production.

[B10-behavsci-16-01477] Duong C. D. (2021). Big Five personality traits and green consumption: Bridging the attitude–intention–behavior gap. Asia Pacific Journal of Marketing and Logistics.

[B11-behavsci-16-01477] Ellen P. S., Wiener J. L., Cobb-Walgren C. (1991). The role of perceived consumer effectiveness in motivating environmentally conscious behaviors. Journal of Public Policy & Marketing.

[B12-behavsci-16-01477] Griskevicius V., Tybur J. M., Van den Bergh B. (2010). Going green to be seen. Journal of Personality and Social Psychology.

[B13-behavsci-16-01477] Haines R., Street M. D., Haines D. (2008). The influence of perceived importance of an ethical issue. Journal of Business Ethics.

[B14-behavsci-16-01477] Hanss D., Böhm G., Doran R., Homburg A. (2016). Sustainable consumption of groceries: The importance of believing that one can contribute to sustainable development. Sustainable Development.

[B15-behavsci-16-01477] He J., Liu K., Ma Z., He Z. (2025). Digital literacy, ecological values, and green food consumption: An extended theory of planned behavior model in Chinese universities. Frontiers in Public Health.

[B16-behavsci-16-01477] He X., Zhan W. (2018). How to activate moral norm to adopt electric vehicles in China?. Journal of Cleaner Production.

[B17-behavsci-16-01477] Hoerl A. E., Kennard R. W. (1970). Ridge regression: Biased estimation for nonorthogonal problems. Technometrics.

[B18-behavsci-16-01477] Jaiswal D., Kant R. (2018). Green purchasing behaviour: A conceptual framework and empirical investigation of Indian consumers. Journal of Retailing and Consumer Services.

[B19-behavsci-16-01477] Jami A., Kouchaki M., Gino F. (2021). I own, so I help out. Journal of Consumer Research.

[B20-behavsci-16-01477] Kinnear T. C., Taylor J. R., Ahmed S. A. (1974). Ecologically concerned consumers: Who are they?. Journal of Marketing.

[B21-behavsci-16-01477] Liang T. C., Situmorang R. O. P., Liao M. C., Chang S. C. (2020). The relationship of perceived consumer effectiveness, subjective knowledge, and purchase intention on carbon label products. Sustainability.

[B22-behavsci-16-01477] Minh T. C., Nguyen T. Q. N. (2024). Factors affecting sustainable consumption behavior: Roles of pandemics and perceived consumer effectiveness. Cleaner and Responsible Consumption.

[B23-behavsci-16-01477] Moscardino U., Miconi D., Calandri E., Altoè G. (2020). Implicit and explicit self-construals, self-esteem and prosocial behavior. Developmental Psychology.

[B24-behavsci-16-01477] Mullainathan S., Spiess J. (2017). Machine learning: An applied econometric approach. Journal of Economic Perspectives.

[B25-behavsci-16-01477] Ng P. M. L., Cheung C. T. Y., Lit K. K., Wan C., Choy E. T. K. (2023). Green consumption and sustainable development: The effects of perceived values and motivation types on green purchase intention. Business Strategy and the Environment.

[B26-behavsci-16-01477] Nguyen H. V., Nguyen C. H., Hoang T. T. B. (2019). Green consumption: Closing the intention–behavior gap. Sustainable Development.

[B27-behavsci-16-01477] Paradise A. W., Kernis M. H. (2002). Self-esteem and psychological well-being. Journal of Social and Clinical Psychology.

[B28-behavsci-16-01477] Patrick H., Neighbors C., Knee C. R. (2004). Appearance-related social comparisons. Personality and Social Psychology Bulletin.

[B29-behavsci-16-01477] Peattie K. (2010). Green consumption: Behavior and norms. Annual Review of Environment and Resources.

[B30-behavsci-16-01477] Plonsky O., Apel R., Ert E., Tennenholtz M., Bourgin D., Peterson J. C., Reichman D., Griffiths T. L., Russell S. J., Carter E. C., Cavanagh J. F., Erev I. (2025). Predicting human decisions with behavioural theories and machine learning. Nature Human Behaviour.

[B31-behavsci-16-01477] Puntoni S., Reczek R. W., Giesler M., Botti S. (2021). Consumers and artificial intelligence: An experiential perspective. Journal of Marketing.

[B32-behavsci-16-01477] Robin D. P., Reidenbach R. E., Forrest P. J. (1996). The perceived importance of an ethical issue. Journal of Business Research.

[B33-behavsci-16-01477] Rosenberg M. (1965). Society and the adolescent self-image.

[B34-behavsci-16-01477] Simmons J. P., Nelson L. D., Simonsohn U. (2011). False-positive psychology: Undisclosed flexibility in data collection and analysis allows presenting anything as significant. Psychological Science.

[B35-behavsci-16-01477] Simonsohn U., Simmons J. P., Nelson L. D. (2020). Specification curve analysis. Nature Human Behaviour.

[B36-behavsci-16-01477] Souiden N., Pons F. (2011). A cross-cultural analysis of consumers’ conspicuous consumption. Journal of International Consumer Marketing.

[B37-behavsci-16-01477] Steegen S., Tuerlinckx F., Gelman A., Vanpaemel W. (2016). Increasing transparency through a multiverse analysis. Perspectives on Psychological Science.

[B38-behavsci-16-01477] Stuppy A., Mead N. L., Van Osselaer S. M. J. (2020). I am, therefore I buy. Journal of Consumer Research.

[B39-behavsci-16-01477] Sun Q., Guo H., Yu X., Zhang W. (2021). More cooperation compensates for lower self-esteem in social dilemmas. Personality and Individual Differences.

[B40-behavsci-16-01477] Tibshirani R. (1996). Regression shrinkage and selection via the Lasso. Journal of the Royal Statistical Society: Series B.

[B41-behavsci-16-01477] Uusitalo O., Rouhiainen N., Salimi N. (2026). Values, risk and biodiversity-friendly action: Perceived consumer effectiveness matters. Journal of Consumer Marketing.

[B42-behavsci-16-01477] Vermeir I., Verbeke W. (2006). Sustainable food consumption: Exploring the consumer attitude–behavioral intention gap. Journal of Agricultural and Environmental Ethics.

[B43-behavsci-16-01477] Vieira V. A., Araújo C. F., Groening C. (2025). The predictor role of perceived consumer effectiveness and environmental concern in consumer green behavior: A meta-analysis with cultural-level moderators. Journal of International Marketing.

[B44-behavsci-16-01477] Xie S., Madni G. R. (2023). Impact of social media on young generation’s green consumption behavior. Sustainability.

[B45-behavsci-16-01477] Yang M., Chen H., Long R., Wang Y. (2023). How does government regulation shape residents’ green consumption behavior?. Journal of Environmental Management.

[B46-behavsci-16-01477] Yaqub M. Z., Yaqub R. M. S., Khan S. Y., Murad M. (2024). How does the government’s sustainable consumption policy enkindle sustainable consumption behaviors in the consumer public?. Cleaner and Responsible Consumption.

[B47-behavsci-16-01477] Zou H., Hastie T. (2005). Regularization and variable selection via the Elastic Net. Journal of the Royal Statistical Society: Series B.

