# Supplementary material for: From Self-Worth to Sustainable Choice: An Efficacy-Translation Account of Green Consumption
Source: Behav Sci (Basel). 2026 Aug 25;16(9):1477. doi: 10.3390/bs16091477 (PMC13603603; doi:10.3390/bs16091477)
Supplement: Supplementary file 1 [file behavsci-16-01477-s001.zip › Supplementary_Materials_S1_Reproducibility_and_Robustness.pdf]

Supplementary Materials

From Self-Worth to Sustainable Choice: An Efficacy-Translation Account of Green Consumption

Tao Jiang, Fengpei Hu, Xiadan Yang, and Ming Guo

Overview

This supplementary file reports reproducibility materials, robustness analyses, and statistical details supporting the main manuscript. The source datasets were harmonized into analysis-ready, study-level datasets, and all estimates and robustness checks were regenerated using the reproducible analysis program. The five studies were not p reregistered. All substantive claims should therefore be read as non-preregistered tests, and the Study 3 issue-importance analysis is exploratory.

S1 Reproducible Analysis Materials

Table S1: Study-level analysis data and core outcomes.

Study	Analysis data	N	Main outcome
Study 1A	Study 1A harmonized data	300	Eight-category trade-off choice
Study 1B	Study 1B harmonized data	299	General green-consumption intention
Study 2A	Study 2A harmonized data	300	Past green-consumption behavior
Study 2B	Study 2B harmonized data	300	Eight-category trade-off choice
Study 3	Study 3 harmonized data	300	General green-consumption intention

S2 Scale Reliability

Most multi-item measures showed acceptable reliability. The Study 1A export contains ten self-esteem items, including four reverse-worded items marked in the source labels; the exported composite uses all ten item-level variables. The questionnaires use a Chinese-language rendering of the ten Rosenberg items. The archive identifies Rosenberg (1965) as the source but does not document a separate validated Chinese translation or adaptation protocol, so the manuscript does not claim use of a specific validated translation. With all ten items included, the lowest self-esteem coefficient was in Study 2A ($\alpha = 0.702$); the lowest PCE coefficient was in Study 3 ($\alpha = 0.622$). The main interpretation therefore emphasizes convergence across studies and robustness checks rather than any single coefficient.

Table S2: Cronbach alpha for detected multi-item scales.

Study	Scale	Items	Alpha
Study 1A	self esteem	10	0.802
Study 1A	green attitude	4	0.739

Study	Scale	Items	Alpha
Study 1A	green value	4	0.872
Study 1A	eight choice	8	0.872
Study 1B	self esteem	10	0.927
Study 1B	green intention	4	0.755
Study 1B	green attitude	4	0.778
Study 1B	green value	4	0.892
Study 2A	self esteem	10	0.702
Study 2A	pce	5	0.711
Study 2A	past green behavior	4	0.702
Study 2A	green attitude	4	0.690
Study 2A	green value	4	0.870
Study 2B	self esteem	10	0.772
Study 2B	pce	5	0.926
Study 2B	green attitude	4	0.926
Study 2B	green value	4	0.936
Study 2B	eight choice	8	0.944
Study 3	self esteem	10	0.732
Study 3	pce	5	0.622
Study 3	green intention	4	0.703
Study 3	green attitude	4	0.719
Study 3	green value	4	0.886
Study 3	issue importance	4	0.702

Descriptive correction-for-attenuation sensitivity analysis

Because measurement error can attenuate correlations, Table S1b rescales the demographic-adjusted partial correlations by the square root of the product of the available self-esteem and outcome reliabilities. These values are descriptive sensitivity estimates, not corrected causal effects. They do not address confounding, common-method bias, non-preregistration, or the fact that reliabilities vary with the operationalization.

Table S3: Descriptive correction-for-attenuation sensitivity analysis.

Study	Observed partial r	α_{SE}	$\alpha_{outcome}$	Corrected r
1A	0.172	0.802	0.872	0.206
1B	0.137	0.927	0.755	0.164
2A	0.167	0.702	0.702	0.237
2B	0.159	0.772	0.944	0.187
3	0.186	0.732	0.703	0.259

S3 Main Effects and Internal Meta-Analysis

S4 Specification-Curve Audit

The specification-curve audit re-estimated the self-esteem effect under four control sets in each study: unadjusted, demographics, green-orientation controls, and demographics plus green-orientation controls. All 20 coefficients were positive, and 18 of 20 reached $p < 0.05$.

Table S4: HC3 robust self-esteem effects by study.

Study	N	Beta	p	CI low	CI high
Study 1A	300	0.153	0.017	0.027	0.279
Study 1B	299	0.088	0.013	0.019	0.158
Study 2A	300	0.129	0.107	-0.028	0.286
Study 2B	300	0.080	0.062	-0.004	0.164
Study 3	300	0.153	0.024	0.020	0.287

Table S5: Leave-one-study-out internal meta-analysis.

Dropped	k	Pooled r	CI low	CI high	I2
Study 1A	4	0.162	0.107	0.217	0.000
Study 1B	4	0.171	0.115	0.226	0.000
Study 2A	4	0.164	0.108	0.219	0.000
Study 2B	4	0.166	0.110	0.220	0.000
Study 3	4	0.159	0.103	0.214	0.000

Table S6: Specification-curve summary.

Study	Specs	Positive	Significant	Median beta	Range
Study 1A	4	1.00	1.00	0.250	[0.153, 0.391]
Study 1B	4	1.00	1.00	0.220	[0.082, 0.360]
Study 2A	4	1.00	0.75	0.302	[0.129, 0.490]
Study 2B	4	1.00	0.75	0.187	[0.080, 0.289]
Study 3	4	1.00	1.00	0.310	[0.138, 0.470]
Overall	20	1.00	0.90	0.230	[0.080, 0.490]

S5 Study 2A and Study 2B Mechanism Checks

Study 2A produced a standardized indirect association of 0.105, 95% bootstrap CI [0.048, 0.164]. The component paths were $a = 0.343$ (HC3 SE = 0.066) and $b = 0.305$ (HC3 SE = 0.069); the total association was $c = 0.129$ (HC3 SE = 0.080, $p = 0.107$) and the direct coefficient was $c' = 0.024$ (HC3 SE = 0.071. Residual diagnostics indicated heteroskedasticity and non-normality, so HC3 standard errors and bootstrap confidence intervals were used. These procedures do not verify the causal assumptions of cross-sectional mediation. Because self-esteem, PCE, and past behavior were measured rather than randomly assigned, this result is consistent with but does not establish causal mediation or full mediation. Study 2B used the same sample for the original simple-slope analysis and the HC3 robust re-estimation; the latter is an alternative specification, not an independent replication.

S5.1 Study 2B Participant-Facing Materials

The updated author-supplied Credamo export and Appendix are archived with this Supplementary Materials file in `Author_Supplied_Study2B_Materials/`. The verified questionnaire sequence was demographics (C1–C6, ten self-esteem items, one randomly presented condition passage with a minimum display time of 18 seconds, five PCE manipulation-check items, four green-consumption-value items, four green-product-attitude items, and eight product choices (E1–E8. The updated export contains no general green-consumption-intention block in Study 2B. Green consumption values and green product

attitude were therefore post-manipulation measures, not baseline measures. The following English translations preserve the participant-facing content.

Low-PCE condition. Participants read: "In recent years, environmental problems have attracted worldwide attention, and governments have called on citizens to engage in green consumption and protect the environment. However, individual capabilities are limited. Even when people respond to the call to improve the environment, their positive impact on society is very small and cannot fundamentally improve environmental conditions. Therefore, people often feel powerless when addressing environmental problems. For example, consumers may hope to protect the environment by purchasing environmentally friendly products, but environmental problems continue to worsen. Many consumers also save water and energy, yet these actions may not solve resource problems, and resources continue to be depleted. In summary, each of us is very small, and it is difficult to improve the environment through our own behavior."

Control condition. Participants read: "Since ancient times, people have tried to find suitable media for recording and transmitting information. From clay tablets and oracle bones to bamboo slips and silk, each medium played an important role during a particular historical period. As civilization developed, however, the limitations of these media became apparent: clay tablets were heavy and fragile, oracle bones were difficult to obtain, bamboo slips occupied too much space, and silk was ex pensive. These limitations made it difficult to meet the growing need for information st orage. Therefore, people often experienced inconvenience when recording and preserving information. Scholars who wished to collect large numbers of classic texts faced piles of bamboo slips that were difficult to read and transport, while government offices found it difficult to copy and distribute official documents quickly. In summary, every early medium had limitations and could not fully meet the expectations of information transmission."

PCE manipulation-check items. The five items were: (1 "I feel that my actions will have a positive impact on alleviating environmental problems"; (2 "By purchasing environmentally friendly products, I am helping to save the environment"; (3 "I believe that my actions can make a difference in solving environmental problems"; (4 "My green consumption will have an impact on solving environmental problems"; and (5 "One person cannot do very much to protect the environment" (reverse scored.

Choice stimuli. The eight two-option trade-offs were as follows. The prices are those displayed in the Credamo export. The archive does not document a separate rationale for selecting these categories.

Table S7: Study 2B eight-category green-versus-conventional choice stimuli.

Category	Option A	Option B
Automobile	Conventional gasoline; RMB 100,000	Pollution-free fuel; RMB 150,000
Light bulb	Conventional material; RMB 8	Environmentally friendly, non-polluting material; RMB 15
Mobile phone	Not recyclable; RMB 1,200	Recyclable; RMB 2,000
Air conditioner	Conventional air conditioner; RMB 2,300	New fluorine-free air conditioner; RMB 3,400
Gasoline	Conventional gasoline; RMB 6.42/litre	Unleaded gasoline; RMB 7.92/litre
Laundry detergent	Conventional detergent; RMB 25/container	No polluting ingredients; RMB 35/container

Milk	Non-recyclable packaging; RMB 3/bottle	Recyclable packaging; RMB 5/bottle
Printing paper	Conventional paper; RMB 20/pack	Recycled paper; RMB 35/pack

The product pairs were not accompanied by separate participant ratings of familiarity or perceived environmental impact. The design therefore confounds the environmental attribute with a price premium and does not permit a claim that the choice score isolates environmental impact alone. The original Chinese questionnaire, screenshots, and measurement Appendix are retained as author-supplied files.

Table S8: Study 2B PCE manipulation check.

Outcome	N control	N low-PCE	M control	M low-PCE	p	d
PCE	150	150	5.536	3.887	<0.001	1.212
G_Eight	150	150	4.930	5.642	<0.001	-0.516

Table S9: Study 2B condition checks for measured constructs.

Construct	M control	M low-PCE	Difference	*p*	*d*	Interpretation
PCE	5.536	3.887	1.649	<0.001	1.212	Intended manipulation check
Self-esteem	35.347	34.920	0.427	0.309	0.118	No detected baseline difference
Green attitude	5.183	6.095	-0.912	<0.001	-0.740	Possible unintended condition difference
Green values	4.573	5.347	-0.773	<0.001	-0.562	Possible unintended condition difference

Table S10: Study 2B self-esteem slope attenuation.

Contrast	N	Condition	SE slope, 95% CI	p	Interaction, 95% CI	p
Full interaction	300	Control vs low-PCE	0.057 [0.021, 0.092]	0.002	-0.044 [-0.099, 0.011]	0.115
Simple slope	150	Control	0.058 [0.022, 0.095]	0.001	–	–
Simple slope	150	Low PCE	0.012 [-0.044, 0.067]	0.680	–	–

No prospective interaction-specific power analysis was available. Conditional on the observed HC3 standard error of approximately 0.028, a two-sided test at $\alpha = 0.05$ would require an interaction of about $|b| = 0.079$ for approximately 80% power. The study was therefore not sufficiently precise to resolve small interaction effects and is described as an inconclusive interaction test with suggestive attenuation.

Study 2B materials and scope of inference

The updated author-supplied files provide the participant-facing manipulation wording, the complete E1–E8 product stimuli, the manipulation-check items, and the verified questionnaire order. The de-identified analytic dataset does not include separate familiarity, environmental-impact, affect, or motivation measures. Because green consumption values and green product attitude were measured after the randomized passage and differed between conditions, those differences may reflect unintended effects rather than baseline imbalance. The available condition means and model re-estimations therefore support a strong PCE manipulation check but cannot establish that the manipulation changed PCE specifically rather than broader motivational or affective responses.

S6 Study 3 Boundary Robustness

The PII boundary evidence is exploratory and non-significant across the reported continuous interaction specifications. The descriptive tertile results and Johnson–Neyman transition points should not be read as evidence of moderation.

Table S11: PII sensitivity analysis.

Scenario	N	SE beta	PII beta	SE x PII	p
full_sample	300	0.070	0.291	-0.043	0.205
remove_PII_gt_6_59	271	0.034	0.337	-0.055	0.073
winsorize_PII_5_95	300	0.096	0.211	-0.044	0.337
PII_tertile_low	158	0.225	–	–	–
PII_tertile_middle	61	0.037	–	–	–
PII_tertile_high	81	0.118	–	–	–

Table S12: Johnson–Neyman transition points for Study 3.

PII z	PII raw	SE slope	SE	t	p
-2.312	4.754	0.169	0.086	1.968	0.050
-1.687	5.099	0.142	0.072	1.968	0.050

S7 Computational Behavioral Audit

The feature set comprised self-esteem, PCE, perceived issue importance, green attitude, green value, three moral-knowledge items, and six demographic variables. Variables were converted to numeric form and complete-case filtering removed no Study 3 records (N = 3 00)Standardization occurred inside each pipeline. The outer evaluation used shuffled five-fold cross-validation (seed 31. Ridge considered 25 log-spaced penalties from 10^{-3} to 10^{3}; Lasso considered 30 penalties from 10^{-3} to 10^{1} with internal five-fold selection; Elastic Net used internal five-fold selection over l1 ratios 0.1, 0.3, 0.5, 0.7, and 0.9. The random forest used 320 trees, minimum leaf size 14, and seed 11. These were reproducible benchmark settings, not an unrestricted hyperparameter search. The permutation-importance model used 220 trees, minimum leaf size 14, and 10 repeats (seed 12. The model benchmark is a secondary descriptive comparison. With N = 300 and multiple predictors, the observed advantage for regularized models may reflect limited data for the random forest to estimate n on-linearities. It does not establish a linear psychological structure.

Table S13: Study 3 model benchmark.

Model	CV R2	SD	MAE	SD
Ridge	0.497	0.087	0.292	0.023
Elastic net	0.489	0.102	0.292	0.020
Lasso	0.482	0.110	0.293	0.018
Linear regression	0.479	0.120	0.293	0.019
Random forest	0.428	0.057	0.304	0.022

Table S14: Study 3 nested ablation audit.

Model family	OLS R2	Delta R2	CV R2	MAE
Demographics	0.056	–	-0.032	0.416
+ Attitude/value/knowledge	0.528	0.473	0.424	0.300
+ Self-esteem	0.543	0.015	0.441	0.296
+ PCE	0.572	0.029	0.462	0.291
+ PII/profile	0.599	0.027	0.502	0.284

S8 Figure Files and Export Quality

The figure assets contain PDF, SVG, PNG, and TIFF versions where available, including the specification-curve figure. The PNG/TIFF figures were exported at 600 dpi. Figure 1 was prepared as a conventional manuscript diagram, with editable PDF/SVG fallbacks retained for alternate journal formats.

S9 Materials Availability and Ethics Timeline

The original Study 2B questionnaire, corrected measurement Appendix, and eight product-category stimuli are archived with this Supplementary Materials file. All five studies were conducted within the approval period (19 October 2025 to 18 October 2026: Study 1A on 6 June 2026, Study 1B on 8 June 2026, Study 2A on 11 June 2026, Study 2B on 15 June 2026, and Study 3 on 18 June 2026. The verified outcome mapping is Study 1B as general green-consumption intention and Study 2A as past green-consumption behavior. Separate familiarity, environmental-impact, affect, and motivation checks were not collected, as detailed in the Study 2B materials section.
