# Supplementary material for: From Self-Worth to Sustainable Choice: An Efficacy-Translation Account of Green Consumption
Source: Behav Sci (Basel). 2026 Aug 25;16(9):1477. doi: 10.3390/bs16091477 (PMC13603603; doi:10.3390/bs16091477)
Supplement: Supplementary file 1 [file behavsci-16-01477-s001.zip › Supplementary_Materials_S2_Study2B_English_Translation_of_Original_Credamo_Questionnaire.pdf]

Supplementary Materials S2: Study 2B English Translation of the Original Credamo Questionnaire (Green Consumption Survey 2B)

Welcome to take part in this quiz!

The survey will be conducted anonymously, and no personal information will be disclosed.

The survey data is intended for scientific research purposes and does not involve any commercial interests.

Please answer based on your genuine feelings .

Thank you again for your time.

Default Block – Section Start

C1 Gender [Single-select] ○ Male ○ Female

C2 Age [Single Choice]

○ Under 18 years old ○ 18–25 years old ○ 26–35 years old ○ 36–45 years old ○ 46–55 years old ○ 56 years old or older

C3 Educational Level [Single Choice]

○ High school or below ○ Junior college/Higher vocational education ○ Bachelor's degree ○ Master's degree ○ Doctoral degree or above

C4 – Monthly Personal Income [Single Choice]

○ RMB 3,000 or less ○ RMB 3,001–5,000 ○ RMB 5,001–8,000

○ RMB 8,001–12,000 ○ RMB 12,001–20,000 ○ RMB 20,000 or more

C5 Marital status and child circumstances [Single-choice]

○ Unmarried ○ Married (no children) ○ Married (with children)

C6 Residence [Single-select] ○ Urban ○ Town ○ Rural

paging

SE1: I feel that I am a valuable person, at least on the same level as others. [Scale]

Doesn't fit well			Very suitable
1<br>○	2<br>○	3<br>○	4<br>○

SE2: I feel that I possess many good qualities. [Scale]

Doesn't fit well			Very suitable
1<br>○	2<br>○	3<br>○	4<br>○

SE3:In the end, I tend to consider myself a failure. [Scale]

Doesn't fit well			Very suitable
1<br>○	2<br>○	3<br>○	4<br>○

SE4: I can do things as well as most people do. [Scale]

Doesn't fit well			Very suitable
1<br>○	2<br>○	3<br>○	4<br>○

SE5: I don't feel that I have many things to be proud of. [Scale]

Doesn't fit well			Very suitable
1<br>○	2<br>○	3<br>○	4<br>○

SE6: I have a positive attitude toward myself. [Scale]

Doesn't fit well			Very suitable
1<br>○	2<br>○	3<br>○	4<br>○

SE7: Overall, I am satisfied with myself. [Scale]

Doesn't fit well			Very suitable
1<br>○	2<br>○	3<br>○	4<br>○

SE8: I hope I can earn more respect for myself. [Scale]

Doesn't fit well			Very suitable
1<br>○	2<br>○	3<br>○	4<br>○

SE9: I often feel like I am of no use at all. [Scale]

Doesn't fit well			Very suitable
1<br>○	2<br>○	3<br>○	4<br>○

SE10: I often feel that I am completely worthless. [Scale]

Doesn't fit well			Very suitable
1<br>○	2<br>○	3<br>○	4<br>○

---

paging

Please read the following passage carefully: (Intervention presented using a between-subjects design with random presentation)

L [Low-PCE Group]

In recent years, environmental issues have drawn widespread attention from people all over the world. Governments have also called on their citizens to engage in green consumption and protect the environment.

However, individual capabilities are limited. Even when people respond to the call to improve the environment, their positive impact on society is very small and cannot fundamentally improve environmental conditions.

Therefore, people often feel powerless when addressing environmental problems. For example, consumers may hope to protect the environment by purchasing environmentally friendly products, but environmental problems continue to worsen. Many consumers also save water and energy, yet these actions may not solve natural resource problems, and resources continue to be depleted. In summary, each of us is very small, and it is difficult to improve the environment through our own behavior.

C [Control Group]

Since ancient times, humanity has continuously sought suitable media for recording and transmitting information. From early clay tablets and oracle bones to later bamboo slips and silk manuscripts, each medium played an important role during its respective historical period.

However, as civilization developed, the limitations of these traditional media gradually became apparent: clay tablets were heavy and fragile; oracle bones were difficult to obtain; bamboo slips occupied too much space; and silk manuscripts were expensive. These limitations made it difficult to meet the growing need for information storage.

As a result, people often feel inconvenienced when recording and preserving information. For example, scholars may wish to organize and preserve large numbers of classic texts, but piles of bamboo slips are difficult to browse and transport. Government offices may also need to transmit official documents, yet these documents are difficult to copy and distribute quickly, so information flow remains severely constrained. In summary, each early medium has its own limitations and cannot fully meet humanity's expectations for information dissemination.

Pagination: Minimum display duration – 18 seconds; countdown display

I feel that my individual actions will have a positive impact on addressing environmental problems. [Scale]

Does not match at all						be completely in conformity with

1<br>○	2<br>○	3<br>○	4<br>○	5<br>○	6<br>○	7<br>○

By purchasing environmentally friendly products, I am helping to protect the environment. [Scale]

Does not match at all						be completely in conformity with
1<br>○	2<br>○	3<br>○	4<br>○	5<br>○	6<br>○	7<br>○

I believe that my actions can make a difference in solving environmental problems. [Scale]

Does not match at all						be completely in conformity with
1<br>○	2<br>○	3<br>○	4<br>○	5<br>○	6<br>○	7<br>○

My green consumption will have an impact on solving environmental problems. [Scale]

Does not match at all						be completely in conformity with
1<br>○	2<br>○	3<br>○	4<br>○	5<br>○	6<br>○	7<br>○

One person cannot do very much to protect the environment. [Scale]

Does not match at all						be completely in conformity with
1<br>○	2<br>○	3<br>○	4<br>○	5<br>○	6<br>○	7<br>○

paging

Buying green products will help me feel accepted by others. [Scale]

Does not match at all						be completely in conformity with
1<br>○	2<br>○	3<br>○	4<br>○	5<br>○	6<br>○	7<br>○

Buying green products will improve others' perception of me. [Scale]

Does not match at all						be completely in conformity with
1<br>○	2<br>○	3<br>○	4<br>○	5<br>○	6<br>○	7<br>○

Buying green products can help me make a positive impression on others. [Scale]

Does not match at all						be completely in conformity with
1<br>○	2<br>○	3<br>○	4<br>○	5<br>○	6<br>○	7<br>○

Buying green products will bring me social recognition. [Scale]

Does not match at all						be completely in conformity with
1<br>○	2<br>○	3<br>○	4<br>○	5<br>○	6<br>○	7<br>○

paging

For me, purchasing green products is [Scale].

bad						good
1<br>○	2<br>○	3<br>○	4<br>○	5<br>○	6<br>○	7<br>○

For me, purchasing green products is [scale].

foolish						wise
1<br>○	2<br>○	3<br>○	4<br>○	5<br>○	6<br>○	7<br>○

For me, purchasing green products is [Scale].

Unpleasant						pleasant
1<br>○	2<br>○	3<br>○	4<br>○	5<br>○	6<br>○	7<br>○

For me, purchasing green products is [scale].

undesirable						desirable
1<br>○	2<br>○	3<br>○	4<br>○	5<br>○	6<br>○	7<br>○

paging

E1: Please choose between the following two products: 1 indicates a complete preference for Product A; 7 indicates a complete preference for Product B.

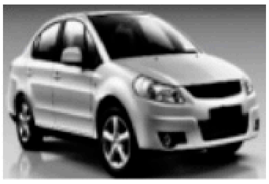

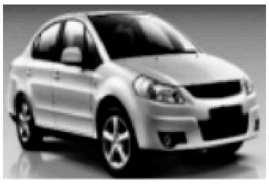

Car A:
Using ordinary gasoline fuel, the price is 100,000 RMB

Car B:
Using non-polluting hydrogen fuel, the price is 150,000 RMB

A						B
1<br>○	2<br>○	3<br>○	4<br>○	5<br>○	6<br>○	7<br>○

E2: Please choose between the following two products: 1 indicates a complete preference for Product A; 7 indicates a complete preference for Product B.

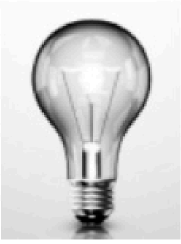

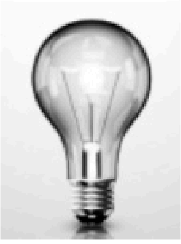

Light bulb A:
Made of ordinary materials, the price is 8 RMB

Light bulb B:
Made of non-polluting materials, price is 15 RMB

A						B
1<br>○	2<br>○	3<br>○	4<br>○	5<br>○	6<br>○	7<br>○

E3: Please choose between the following two products: 1 indicates a complete preference for Product A; 7 indicates a complete preference for Product B.

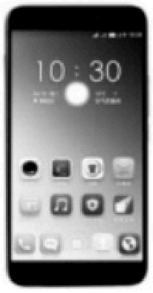

Cell phone A:
Product is not recyclable, the price is 1200 RMB

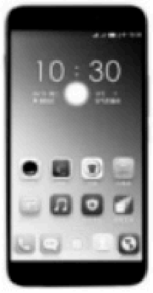

Cell phone B:
Product can be recycled, the price is 2,000 RMB

A						B
1<br>○	2<br>○	3<br>○	4<br>○	5<br>○	6<br>○	7<br>○

E4: Please choose between the following two products: 1 indicates a complete preference for Product A; 7 indicates a complete preference for Product B.

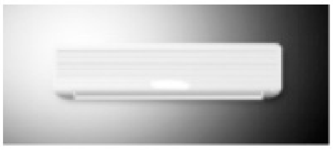

Air conditioner A:
Ordinary air conditioner, the price is 2300 RMB

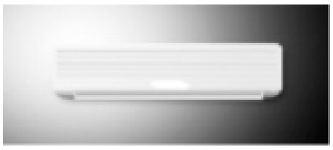

Air conditioner B:
New fluorine-free air conditioner, price is 3400 RMB

A						B
1<br>○	2<br>○	3<br>○	4<br>○	5<br>○	6<br>○	7<br>○

E5: Please choose between the following two products: 1 indicates a complete preference for Product A; 7 indicates a complete preference for Product B.

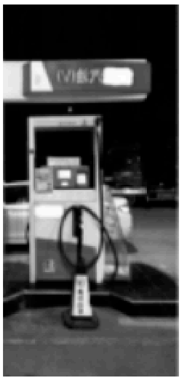

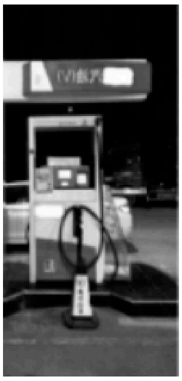

Gasoline A:
Ordinary gasoline, price is 6.42 RMB/liter

Gasoline B:
Unleaded gasoline, the price is 7.92 RMB/liter

A						B
1<br>○	2<br>○	3<br>○	4<br>○	5<br>○	6<br>○	7<br>○

E6: Please choose between the following two products: 1 indicates a complete preference for Product A; 7 indicates a complete preference for Product B.

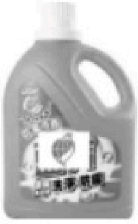

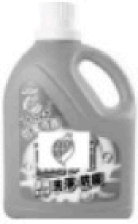

Detergent A:
Ordinary detergent, the price is 25 RMB / barrel

Detergent B:
No polluting ingredients, the price is 35 RMB/barrel

A						B
1<br>○	2<br>○	3<br>○	4<br>○	5<br>○	6<br>○	7<br>○

E7: Please choose between the following two products: 1 indicates a complete preference for Product A; 7 indicates a complete preference for Product B.

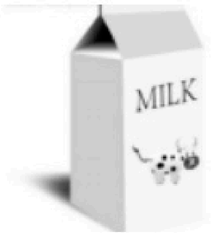

Milk A:
Packaging is not recyclable, the price is 3 RMB/bottle

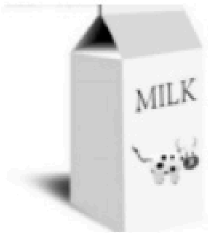

Milk B:
Packaging can be recycled, the price is 5 RMB/bottle

A						B
1<br>○	2<br>○	3<br>○	4<br>○	5<br>○	6<br>○	7<br>○

E8: Please choose between the following two products: 1 indicates a complete preference for Product A; 7 indicates a complete preference for Product B.

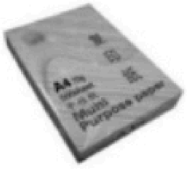

Printing paper A:
Ordinary paper, the price is 20 RMB / pile

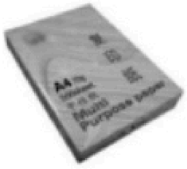

Printing paper B:
Recycled paper, the price is 35 RMB / pile

A						B
1<br>○	2<br>○	3<br>○	4<br>○	5<br>○	6<br>○	7<br>○
