# Supplementary material for: From Self-Worth to Sustainable Choice: An Efficacy-Translation Account of Green Consumption
Source: Behav Sci (Basel). 2026 Aug 25;16(9):1477. doi: 10.3390/bs16091477 (PMC13603603; doi:10.3390/bs16091477)
Supplement: Supplementary file 1 [file behavsci-16-01477-s001.zip › Supplementary_Materials_S2_Study2B_Original_Chinese_Questionnaire.pdf]

Supplementary Materials S2: Study 2B Original Chinese Credamo Questionnaire

欢迎参与此次作答。

调查将采用匿名的方式，不会透漏任何私人信息。

调查数据为科研所用，不涉及任何商业利益。

请根据自己的真实感受，放心作答。

再次感谢您的时间。

默认块-版块开始

C1 性别［单选］ ○ 男 ○ 女

C2 年龄［单选］

○ 18岁及以下○ 18－25岁○ 26－35岁○ 36－45岁○ 46－55岁○ 56岁及以上

C3 受教育程度[单选]

○ 高中及以下 ○ 大专/高职 ○ 本科 ○ 硕士 ○ 博士及以上

C4 个人月收入[单选]

○ 3,000元及以下 ○ 3,001－5,000元 ○ 5,001－8,000元

○ 8,001－12,000元 ○ 12,001－20,000元 ○ 20,000元以上

C5 婚姻状况与子女情况[单选]

○ 未婚 ○ 已婚（无子女） ○ 已婚（有子女）

C6 居住地［单选］ ○ 城市 ○ 城镇 ○ 农村

分页

SE1 我感到我是一个有价值的人，至少与其他人在同一水平上。[量表]

很不符合			非常符合
1<br>○	2<br>○	3<br>○	4<br>○

SE2 我感到我有许多好的品质。[量表]

很不符合			非常符合
1<br>○	2<br>○	3<br>○	4<br>○

SE3 归根结底，我倾向于觉得自己是一个失败者。[量表]

很不符合			非常符合
1<br>○	2<br>○	3<br>○	4<br>○

SE4 我能像大多数人一样把事情做好。[量表]

很不符合			非常符合
1<br>○	2<br>○	3<br>○	4<br>○

SE5 我感到自己值得自豪的地方不多。[量表]

很不符合			非常符合
1<br>○	2<br>○	3<br>○	4<br>○

SE6 我对自己持肯定态度。[量表]

很不符合			非常符合
1<br>○	2<br>○	3<br>○	4<br>○

SE7 总的来说，我对自己是满意的。[量表]

很不符合			非常符合
1	2	3	4

○	○	○	○

SE8 我希望我能为自己赢得更多尊重。[量表]

很不符合			非常符合
1<br>○	2<br>○	3<br>○	4<br>○

SE9 我确实时常感到自己毫无用处。[量表]

很不符合			非常符合
1<br>○	2<br>○	3<br>○	4<br>○

SE10 我时常认为自己一无是处。[量表]

很不符合			非常符合
1<br>○	2<br>○	3<br>○	4<br>○

分页

请认真阅读下面这段话：（被试间设计随机呈现）

L [低启动组]

近年来环境问题引起了全世界人民的关注，各国政府也纷纷呼吁本国人民进行绿色消费、保护环境。但个人的能力毕竟有限，即使响应“改善环境”的呼吁，人们对社会产生的积极影响也非常小，难以从根本上改善环境。

因此很多时候，人们对解决环境问题感到无能为力。比如消费者希望通过购买环境友好型产品来保护环境，但现实是环境问题一直在加剧；又比如很多消费者节约用水和能源，但也难以帮助解决自然资源问题，资源仍不断被损耗，面临枯竭。

综上，其实我们每个人都是非常渺小的存在，很难通过自己的行为来改善环境。

C［控制组］

自古以来，人类就不断尝试寻找合适的载体来记录和传递信息。从早期的泥板、甲骨，到后来的竹简、绢帛，每一种载体都在特定历史阶段发挥了重要作用。但随着文明的发展，这些传统载体的局限性也逐渐显现：泥板笨重易碎，甲骨取材不易，竹简占用空间过大，绢帛成本高昂，难以满足日益增长的信息存储需求。

因此很多时候，人们在记录和保存信息时感到不便。比如学者们希望将大量典籍整理收藏，但现实是竹简堆积如山，翻阅和运输都极为困难；又比如官府需要传递政令文书，但也难以快速复制分发，信息的流通仍然受到严重制约。

综上，其实每一种早期载体都存在自身的缺陷，很难匹配人类对信息传播的全部期待。

分页：最短显示18秒，显示倒计时

PCE1 我感到自己的行为对缓解环境问题将产生积极影响。[量表]

完全不符合						完全符合
1<br>○	2<br>○	3<br>○	4<br>○	5<br>○	6<br>○	7<br>○

PCE2 通过购买环境友好型产品，我正在帮助拯救环境。[量表]

完全不符合						完全符合
1<br>○	2<br>○	3<br>○	4<br>○	5<br>○	6<br>○	7<br>○

PCE3 我认为我的行动能对解决环境问题有所作为。[量表]

完全不符合						完全符合
1<br>○	2<br>○	3<br>○	4<br>○	5<br>○	6<br>○	7<br>○

PCE4 我的绿色消费行为将对解决环境问题产生影响。[量表]

完全不符合						完全符合

1<br>○	2<br>○	3<br>○	4<br>○	5<br>○	6<br>○	7<br>○

PCE5 单靠我一个人对环境保护作用不大。[量表]

完全不符合						完全符合
1<br>○	2<br>○	3<br>○	4<br>○	5<br>○	6<br>○	7<br>○

分页

SV1 购买绿色产品会帮助我感觉被他人接受。[量表]

完全不符合						完全符合
1<br>○	2<br>○	3<br>○	4<br>○	5<br>○	6<br>○	7<br>○

SV2 购买绿色产品会改善他人对我的看法。[量表]

完全不符合						完全符合
1<br>○	2<br>○	3<br>○	4<br>○	5<br>○	6<br>○	7<br>○

SV3 购买绿色产品能帮助我在他人面前留下良好印象。[量表]

完全不符合						完全符合
1<br>○	2<br>○	3<br>○	4<br>○	5<br>○	6<br>○	7<br>○

SV4 购买绿色产品会为我带来社会认可。[量表]

完全不符合						完全符合
1<br>○	2<br>○	3<br>○	4<br>○	5<br>○	6<br>○	7<br>○

分页

GA1 对我而言，购买绿色产品是[量表]

有害的						有益的
1<br>○	2<br>○	3<br>○	4<br>○	5<br>○	6<br>○	7<br>○

GA2 对我而言，购买绿色产品是[量表]

不明智的						明智的
1<br>○	2<br>○	3<br>○	4<br>○	5<br>○	6<br>○	7<br>○

GA3 对我而言，购买绿色产品是[量表]

不愉悦的						愉悦的
1<br>○	2<br>○	3<br>○	4<br>○	5<br>○	6<br>○	7<br>○

GA4 对我而言，购买绿色产品是[量表]

不喜欢的						喜欢的
1<br>○	2<br>○	3<br>○	4<br>○	5<br>○	6<br>○	7<br>○

分页

E1　请在以下两个产品之间做出选择：1代表完全偏向A，7代表完全偏向B

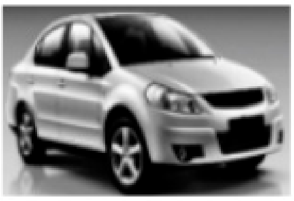

汽车 A
使用普通汽油燃料
价格：10万元人民币

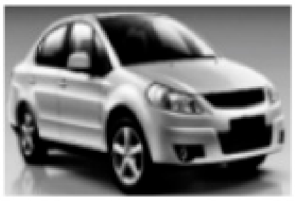

汽车 B
使用无污染的氢燃料
价格：15万元人民币

A						B
1<br>○	2<br>○	3<br>○	4<br>○	5<br>○	6<br>○	7<br>○

E2　请在以下两个产品之间做出选择：1代表完全偏向A，7代表完全偏向B

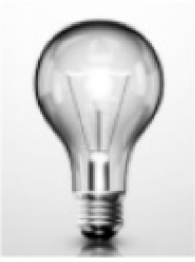

灯泡 A
普通材质
价格：8元

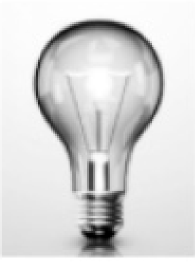

灯泡 B
材质环保无污染
价格：15元

A						B
1<br>○	2<br>○	3<br>○	4<br>○	5<br>○	6<br>○	7<br>○

E3　请在以下两个产品之间做出选择：1代表完全偏向A，7代表完全偏向B

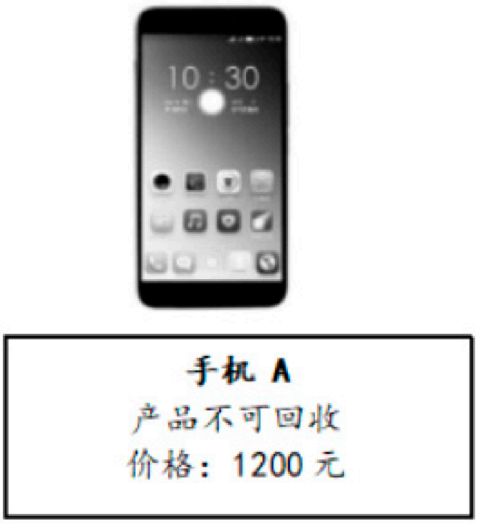

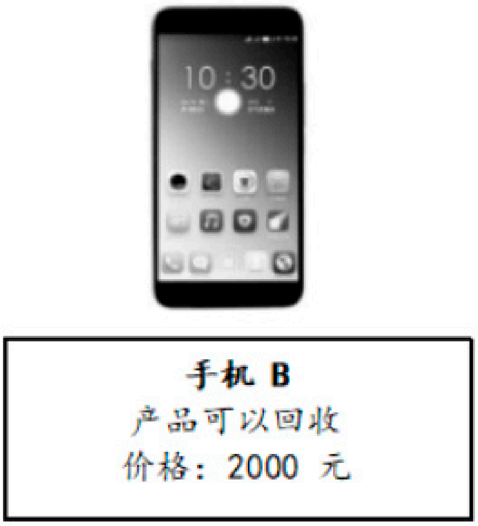

A						B
1<br>○	2<br>○	3<br>○	4<br>○	5<br>○	6<br>○	7<br>○

E4　请在以下两个产品之间做出选择：1代表完全偏向A，7代表完全偏向B

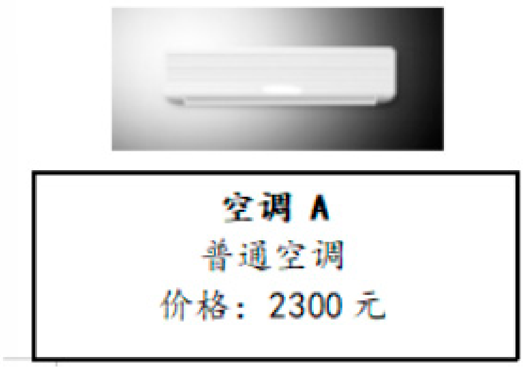

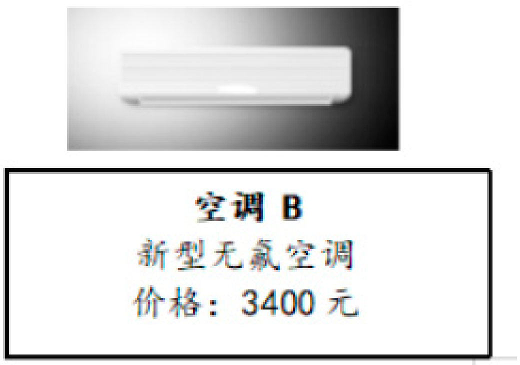

A						B
1<br>○	2<br>○	3<br>○	4<br>○	5<br>○	6<br>○	7<br>○

E5　请在以下两个产品之间做出选择：1代表完全偏向A，7代表完全偏向B

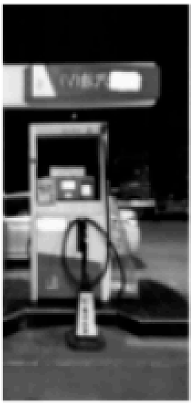

汽油 A
普通汽油
价格：6.42 元/升

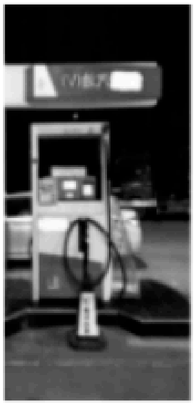

汽油 B
无铅汽油
价格：7.92 元/升

A						B
1 ○	2 ○	3 ○	4 ○	5 ○	6 ○	7 ○

E6　请在以下两个产品之间做出选择：1代表完全偏向A，7代表完全偏向B

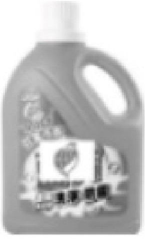

洗涤剂 A
普通洗涤剂
价格：25 元/桶

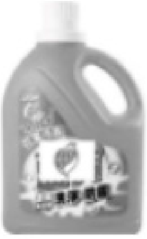

洗涤剂 B
不含污染成分
价格：35 元/桶

A						B
1 ○	2 ○	3 ○	4 ○	5 ○	6 ○	7 ○

E7　请在以下两个产品之间做出选择：1代表完全偏向A，7代表完全偏向B

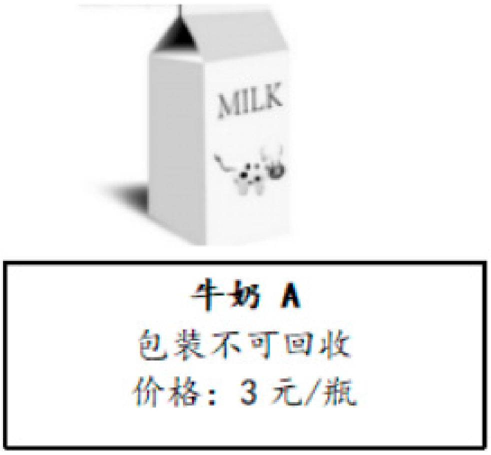

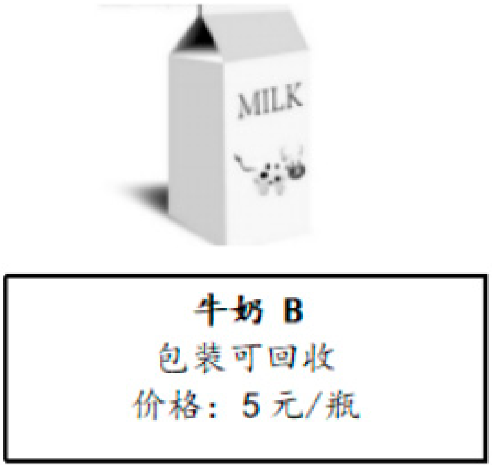

A						B
1<br>○	2<br>○	3<br>○	4<br>○	5<br>○	6<br>○	7<br>○

E8　请在以下两个产品之间做出选择：1代表完全偏向A，7代表完全偏向B

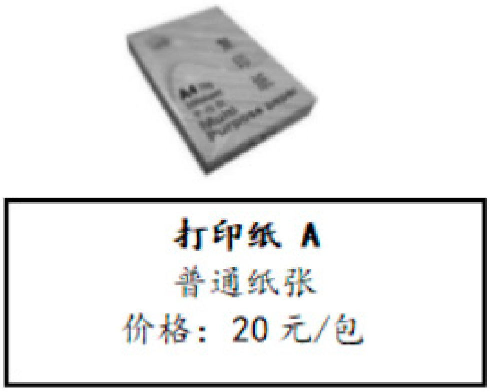

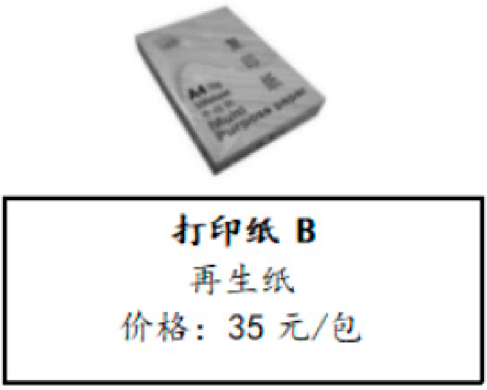

A						B
1<br>○	2<br>○	3<br>○	4<br>○	5<br>○	6<br>○	7<br>○
