# Supplementary material for: From Self-Worth to Sustainable Choice: An Efficacy-Translation Account of Green Consumption
Source: Behav Sci (Basel). 2026 Aug 25;16(9):1477. doi: 10.3390/bs16091477 (PMC13603603; doi:10.3390/bs16091477)
Supplement: Supplementary file 1 [file behavsci-16-01477-s001.zip › Supplementary_Materials_S3_Study2B_English_Measurement_Materials.pdf]

Supplementary Materials S3: Study 2B English

Measurement Materials

S1.Eight-category green versus conventional product choices - study 1A and 2B

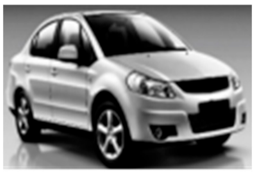

Car A:
Using ordinary gasoline fuel, the price is 100,000 RMB

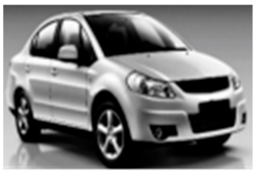

Car B:
Using non-polluting hydrogen fuel, the price is 150,000 RMB

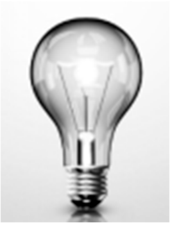

Light bulb A:
Made of ordinary materials, the price is 8 RMB

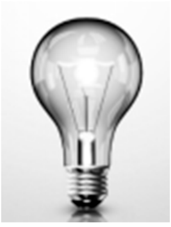

Light bulb B:
Made of non-polluting materials, price is 15 RMB

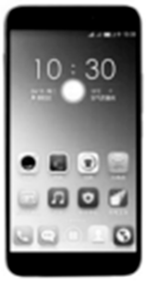

Cell phone A:
Product is not recyclable, the price is 1200 RMB

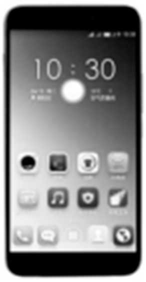

Cell phone B:
Product can be recycled, the price is 2,000 RMB

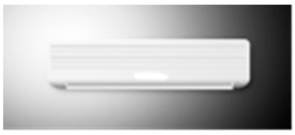

Air conditioner A:
Ordinary air conditioner, the price is 2300 RMB

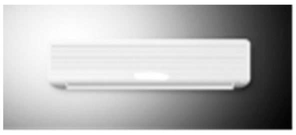

Air conditioner B:
New fluorine-free air conditioner, price is 3400 RMB

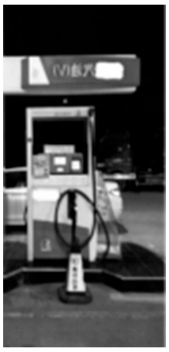

Gasoline A:
Ordinary gasoline, price is 6.42 RMB/liter

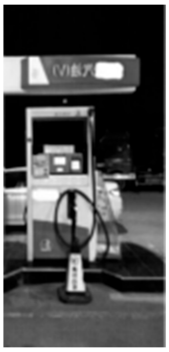

Gasoline B:
Unleaded gasoline, the price is 7.92 RMB/liter

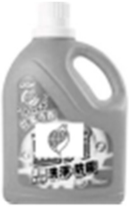

Detergent A:
Ordinary detergent, the price is 25 RMB / barrel

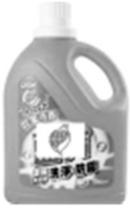

Detergent B:
No polluting ingredients, the price is 35 RMB/barrel

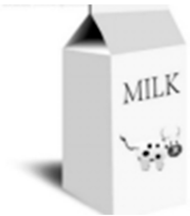

Milk A:
Packaging is not recyclable, the price is 3 RMB/bottle

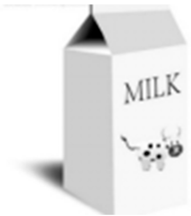

Milk B:
Packaging can be recycled, the price is 5 RMB/bottle

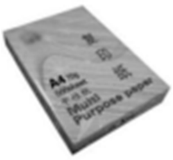

Printing paper A:
Ordinary paper, the price is 20 RMB / pile

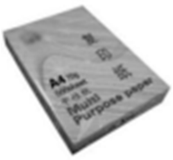

Printing paper B:
Recycled paper, the price is 35 RMB / pile

S2.Past green consumption behavior - study 2A

1	Over the past year, I have purchased environmentally friendly products.	Straughan & Roberts (1999)
2	I often purchase green/eco-friendly products.	
3	I always give priority to choosing environmentally friendly products when I am shopping.	
4	I have reduced the purchase of products that may cause environmental pollution.	

S3.General green consumption intention - study 1B and 3

1	I am willing to purchase products that are beneficial to the environment.	Sheng et al. (2021)
2	I will consider purchasing green products.	
3	I plan to purchase green products in the future.	
4	I am very likely to purchase green products in the future.	

S4.Perceived consumer effectiveness (PCE)

(1)Measurement of perceived consumer effectiveness (study 2A /2B/3)

1	I feel the positive impact that my individual actions will have in preserving environmental problems	Vermeir &Verbeke (2006)
2	By purchasing environmentally friendly products,I am helping to save the environment	
3	I believe that my actions can make a difference in solving environmental problems	
4	My green consumption will have an impact on solving environmental problems.	
5	One person cannot do very much to protect the environment. (R)	

(2)Manipulation of low perceived consumer effectiveness (study 2B)

In recent years, environmental issues have drawn widespread attention from people all over the world. Governments have also called on their citizens to engage in green consumption and protect the environment. However, the capabilities of individuals are limited. Even if they respond to the call to "improve the environment", the positive impact they have on society is very small and it is difficult to fundamentally improve the environment. Therefore, in many cases, people feel powerless in addressing environmental problems. In conclusion, in fact, each of us is a very insignificant being and it is difficult to

improve the environment through our own actions.

S5. Manipulation of Self-esteem (study 1B)

(1) Manipulating low self-esteem

Please recall a very unsuccessful event that you have experienced. In this event, you once doubted your own abilities and felt that you were useless and of no value.

Try to recall the details of the event as much as possible, and complete all the tasks with the feelings you had at that time.

Please describe the cause of this event in 2 to 4 sentences (more than 30 words):

Please describe in 2 to 4 sentences what you did in this matter (more than 30 words):

Please summarize the outcome of this matter in 30 words or more, using 2 to 4 sentences.

Please describe the impact this event has had on you in 2 to 4 sentences (more than 30 words):

(2) Manipulating high self-esteem

Please recall a very successful thing you have done. In this event, you discovered your own strong potential and abilities, and felt that you were a very useful and valuable person.

Try to recall the details of the event as much as possible, and complete all the tasks with the feelings you had at that time.

Please describe the cause of this event in 2 to 4 sentences (more than 30 words):

Please describe in 2 to 4 sentences what you did in this matter (more than 30 words):

Please summarize the outcome of this matter in 30 words or more, using 2 to 4 sentences.

Please describe the impact this event has had on you in 2 to 4 sentences (more than 30 words):

S6.Self-esteem items (study 1A/1B/2A/2B/3)

1	I feel that I am a valuable person, at least on the same level as others.	Rosenberg (1965)
2	I feel that I possess many good qualities.	
3	In the end, I tend to consider myself a failure. (R)	
4	I can do things well like most people do.	
5	I don't feel that I have many things to be proud of. (R)	
6	I have a positive attitude towards myself.	
7	Overall, I am satisfied with myself.	
8	I hope I can earn more respect for myself.	
9	I do often feel that I am of no use at all. (R)	
10	I often think that I am completely worthless. (R)	

S7. Green products attitude

1	purchasing green products is: bad(1) – good (7).	Kim et al. (2026)
2	purchasing green products is: foolish (1) – wise (7).	
3	purchasing green products is: unpleasant (1) – pleasant (7).	
4	purchasing green products is: undesirable (1) – desirable (7).	

S8.Green consumption values

1	Buying green products will help me feel accepted by others.	Koller et al. (2011); Sweeney & Soutar (2001)
2	Buying green products will improve others' perception of me.	
3	Buying green products can help me make a positive impression on others.	
4	Buying green products will bring me social recognition.	
